# Dedicated and Reconfigurable Artificial Neurons and Synapses based on Two-Dimensional Materials for Efficient Neuromorphic Application

**DOI:** 10.1007/s40820-026-02139-2

**Published:** 2026-03-31

**Authors:** Danke Chen, Peizhi Yu, Yuning Li, Jingwei Shang, Haoyuan Wu, Xuan Yao, Xiaoqiu Tang, Chunlong Li, Mingqiang Zhu, Chang Gao, Jingye Sun, He Tian, Tao Deng

**Affiliations:** 1 State Key Laboratory of Advanced Rail Autonomous Operation, Beijing Jiaotong University, Beijing, 100044 People’s Republic of China; 2 School of Electronic and Information Engineering, Beijing Jiaotong University, Beijing, 100044 People’s Republic of China; 3 Department of Precision Instrument, Tsinghua University, Beijing, 100049 People’s Republic of China; 4 School of Integrated Circuits and Beijing National Research Center for Information Science and Technology (BNRist), Tsinghua University, Beijing, 100049 People’s Republic of China

**Keywords:** Two-dimensional materials, Artificial neurons, Artificial synapses, Brain simulation, Reconfigurability

## Abstract

Neuromorphic computing, a highly promising computational architecture, has provided an efficient solution to overcome the limitations of storage–compute separation and scaling constraints. The key to implementing this architecture lies in the development of artificial neurons and synapses as core neuromorphic components capable of biomimicry. Diverse libraries of two-dimensional (2D) materials with atomic-scale thickness and rich tunable physicochemical properties have risen to prominence in recent years. These unique properties meet the critical requirements of neuromorphic devices for ultralow power consumption, dynamic plasticity, and multifunctional integration, thereby facilitating breakthroughs in next-generation high-performance and versatile neuromorphic hardware systems. In this paper, recent advances in dedicated artificial neuron and synapse devices based on 2D materials are reviewed, with a focus on biomimetic models, physical mechanisms, and performance metrics. The discussion further extends to sophisticated switching strategies in reconfigurable components. Then, the systemic integration of neuromorphic devices is summarized, with particular focus on their functional roles in neural perception, neural networks, and logical operation tasks. Finally, a systematic analysis of the limitations at the device and system levels for artificial neurons and synapses is presented, charting a roadmap toward more efficient and multifunctional brain-like chips.

## Highlights

- This review summarizes recent advances in two-dimensional materials-based artificial neurons and synapses, focusing on their biomimetic models, physical mechanisms, and performance metrics, and further discusses sophisticated switching strategies in reconfigurable components.
- The systemic integration of neuromorphic devices is presented, with particular emphasis on their functional roles in perception, neural networks, and logical operation tasks.
- A holistic analysis of the challenge in developing artificial neuronal and synaptic devices and systems is presented, charting a roadmap toward more efficient and multifunctional brain-like chips.

## Graphical abstract

**Figure:**
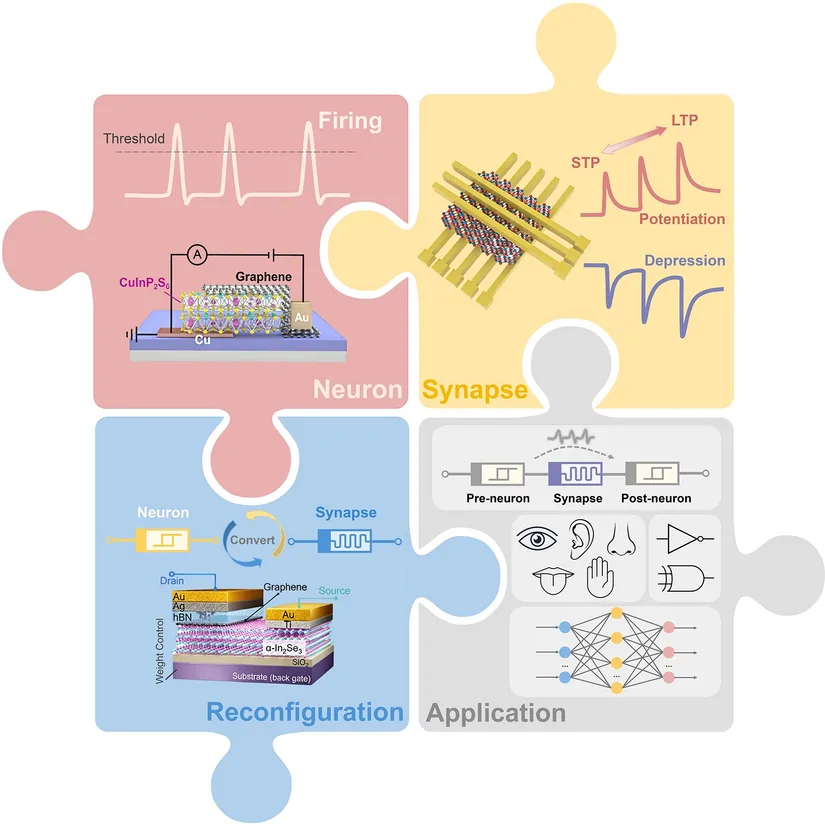

## Introduction

Currently, computing systems are primarily based on the von Neumann architecture, which features the shared storage of instructions and data in memory units, with execution by a central processing unit [1]. However, such a memory–computation separated architecture introduces severe communication bottlenecks, exhibiting progressively exacerbated inherent limitations during large-scale data processing, notably inefficient execution, constrained throughput, and excessive energy consumption [2, 3]. Moreover, as Moore’s law gradually approaches the scaling limits of silicon-based processes, hardware systems are confronting fundamental physical constraints such as short-channel effects, quantum tunneling, and thermal dissipation [4, 5]. These challenges are compounded by the exponentially rising manufacturing costs and the performance saturation of conventional semiconductor materials. The dual dilemma of architectural limitations and process bottlenecks presents fundamental technical challenges for Artificial Intelligence and Internet of Things (AIoT) systems in real-time information processing, energy efficiency optimization, and system scalability. To propel technological innovation in microelectronics, the International Technology Roadmap for Semiconductors has outlined three pivotal pathways: More Moore, More than Moore, and Beyond CMOS (Complementary Metal–Oxide–Semiconductor). The three technological directions are, respectively, dedicated to the continuous miniaturization of transistors, heterogeneous integration for functional diversification, and breakthroughs in novel device and information processing technologies. Particularly, the Beyond CMOS pathway, focused on disruptive technologies to supplement or supersede silicon CMOS, is garnering considerable attention. A central research thrust within this pathway is the exploration of emerging device–architecture interactions utilizing novel materials, which presents promising strategies for addressing the challenges of the architectural bottleneck and scaling limits.

Neuromorphic computing (NC), a groundbreaking computational architecture in the Beyond CMOS roadmap, seeks to compensate for the flaws of conventional architectures by emulating the structural characteristics and biological mechanisms of biological nervous systems [6, 7]. In terms of hardware, the essence of NC lies in the physical mapping of bio-neuromorphic key components while preserving sufficiently plausible dynamics of neural systems. Artificial neurons and synapses serve as fundamental building blocks for artificial neural systems, enabling accurate emulation of biological information processing behavior. As research deepens, neuromorphic devices (based on memristors, memtransistors, memcapacitors, etc.) with dynamic characteristics are progressively replacing early-generation complex CMOS circuits [8, 9], enabling more compact hardware-level integration of information encoding, transmission, processing, and storage. Neuron devices integrate inputs through threshold-triggered electrical responses to generate informative spike trains. Subsequent cascading with synaptic devices leverages non-volatility for concurrent data storage and processing. Such devices designed for NC tasks meet the stringent demands of critical applications requiring high reliability and real-time decisions. Meanwhile, inspired by the dynamic resource allocation concepts in traditional reconfigurable computing architectures (e.g., Field-Programmable Gate Array, FPGA, and Coarse-Grained Reconfigurable Array, CGRA), reconfigurable neuromorphic devices (RNDs) have been developed [10, 11]. These devices enable biomimetic fusion of synaptic and neuronal functionalities on identical hardware platforms through adaptive switching. RNDs exhibit material homology and structural isomorphism, which maximizes the utilization of their intrinsic advantages while preventing potential resource mismatches or shortages. They demonstrate excellent adaptability to the requirements of system integration, high-efficiency operation, and intelligent functionality.

The development of energy-efficient and highly integrated neuromorphic systems necessitates the exploration of novel materials with both dimensional scaling and excellent performance. Emerging two-dimensional (2D) materials offer a promising material platform for the Beyond CMOS technologies. With electronic properties ranging from insulating (e.g., h-BN) and semiconducting (e.g., transition metal dichalcogenides, black phosphorus) to semimetallic (e.g., graphene) and superconducting (e.g., NbSe_2_), the 2D material library provides versatile options for neuromorphic engineering. Materials with specific electrical conductivity, bandgap, and carrier mobility can be selected according to the requirements of different neuromorphic functions. For instance, h-BN can serve as a gate dielectric or passivation layer; semiconductors such as MoS_2_ constitute the functional layer for conductivity modulation, while high-mobility materials like graphene are suitable for use as high-speed carrier transport channels or electrodes. Additionally, the sub-nanometer thickness (such as the 0.335 nm thickness of monolayer graphene) of 2D materials offers exceptional electrostatic control, effectively suppressing short-channel effects, thereby enabling a high on–off ratio and further supporting device scaling alongside a significant reduction in the operating power consumption. Moreover, the 2D materials exhibit rich and tunable physical properties. Materials including WSe_2_, In_2_Se_3_, and 2D perovskite exhibit inherent responsiveness to multiple stimuli (e.g., electrical, optical, mechanical, thermal), allowing them to replicate the multimodal sensing capabilities of biological sensory organs. And the key physical parameters, such as band gap, work function, carrier type, and even lattice structure, can be designed and modulated by the number of layers, strain, electric field, doping, interface engineering, or stacking angle, which endows the device with both plasticity and reconfigurable neuromorphic functionality. Last but not least, the dangling bond-free surfaces of 2D materials facilitate van der Waals integration without lattice matching requirements. Such “Lego-like” modular assembly strategy establishes a material foundation for developing multifunctional neuromorphic systems. Overall, neuromorphic hardware founded on 2D materials presents distinctive prospects for addressing existing technical limitations and pioneering the evolution of microelectronics beyond the Moore’s law era.

**Fig. 1:**
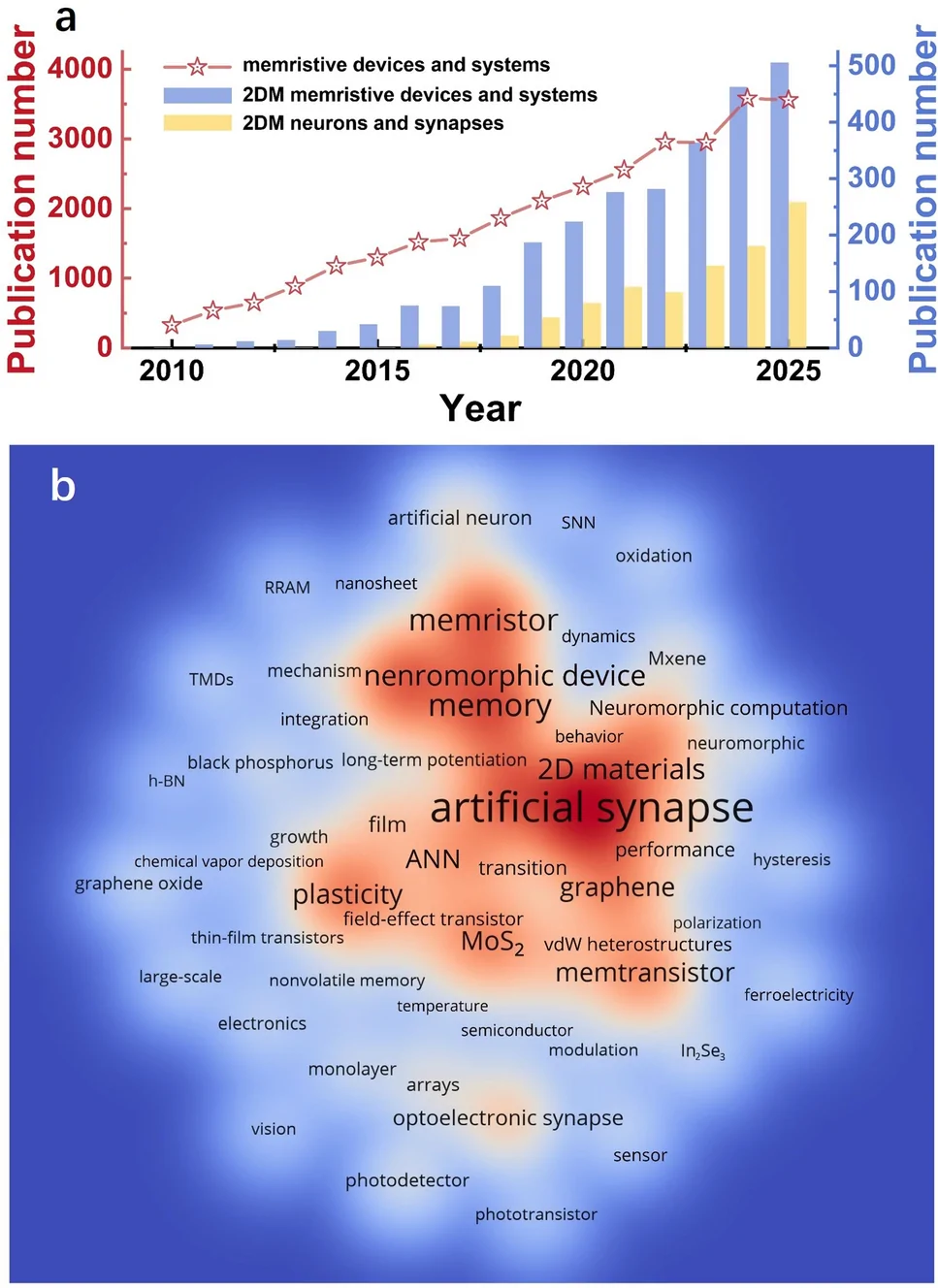
Bibliometric analysis. **a** Annual publication trend over the past 15 years in memristive devices and systems, 2D material-based (2DM) memristive devices and systems, and 2DM artificial neurons and synapses. **b** Landscape of 2DM artificial neurons and synapses: a keyword heatmap analysis

For a comprehensive analysis of research trends and emerging hotspots, a bibliometric analysis was conducted based on the Web of Science Core Collection. The annual publication trend over the past 15 years is presented in Fig. 1a. The robust upward trend in publications on memristive devices and systems demonstrates their rise to prominence as a global research focal point. Driven by the urgent need for novel hardware in integrated circuits and artificial intelligence (AI), research on novel 2D material-based memristive devices and systems and 2D material-based neurons and synapses has increased by a factor of 2.7 and 4.8 since 2019. Figure 1b presents a keyword heatmap based on the literature concerning 2D material-based artificial neurons and synapses. The co-occurrence analysis of keywords from extensive publications visually reveals the current research focus and the overall research landscape. The hotspots primarily converge on material properties, electronic devices, performances, and neural network computing. This indicates that a complete and closely integrated research system is now preliminarily in place. Future research is expected to build upon the foundation of comprehensive exploration and validation to make progress toward more efficient brain-inspired intelligence.

**Fig. 2:**
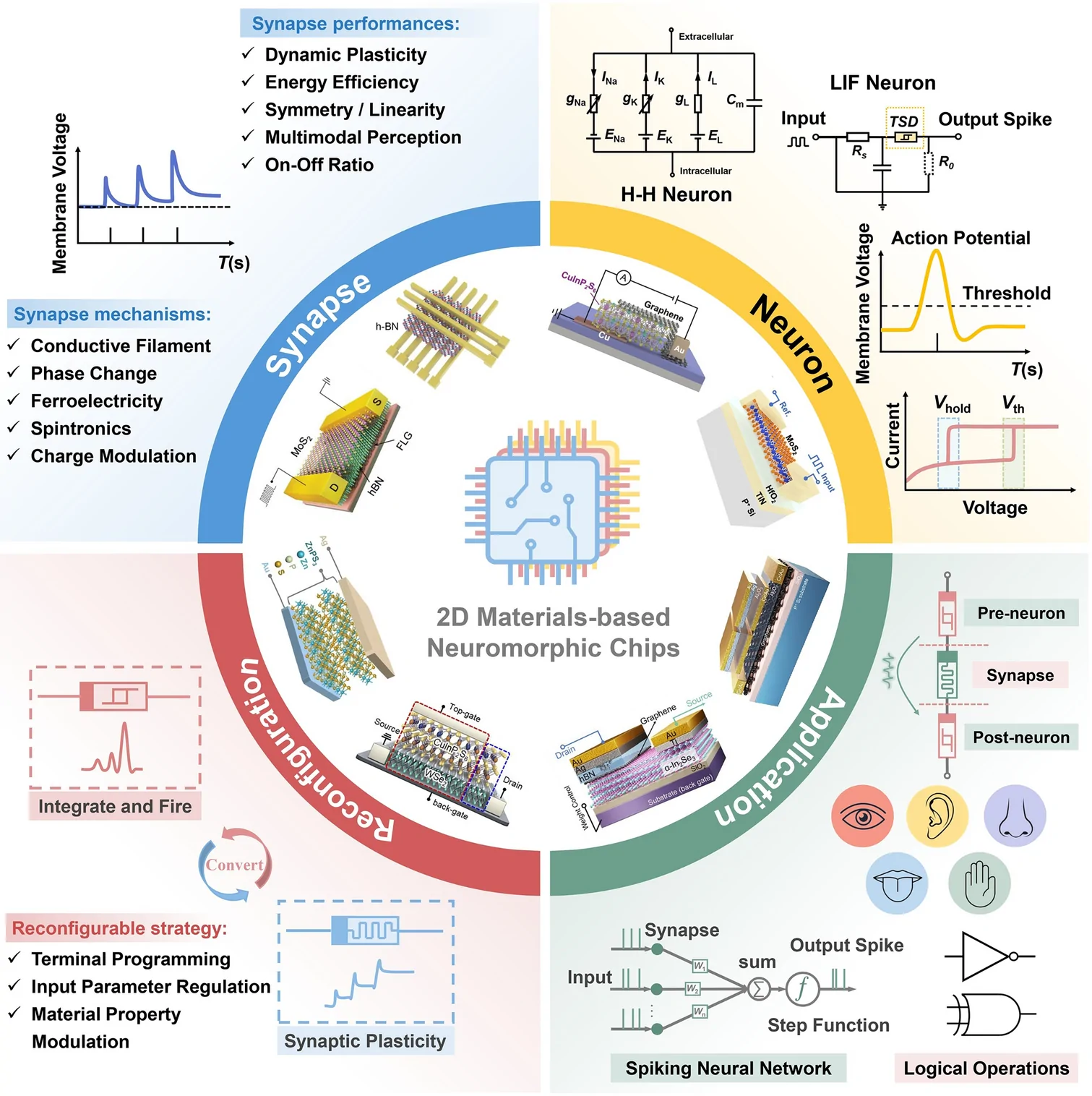
Summary of the reports on dedicated and reconfigurable artificial neurons and synapses, including the biomimetic models, physical mechanisms, dynamic behaviors, reconfigurable strategies, and applications. Schematic illustrations of the representative 2D material-based devices including synapse devices (Reproduced with permission [12]. Copyright (2021), Wiley–VCH GmbH. Reproduced with permission [13]. Copyright (2023), Royal society of chemistry.), neuron devices (Reproduced with permission [14]. Copyright (2023), American Chemical Society. Reproduced with permission [15]. Copyright (2023), American Chemical Society.), reconfigurable devices (Reproduced with permission [16]. Copyright (2025), Springer Nature. Reproduced with permission [17]. Copyright (2025), Wiley–VCH GmbH.), and applications (Reproduced with permission [18]. Copyright (2025), Wiley–VCH GmbH. Reproduced with permission [19]. Copyright (2024), Wiley–VCH GmbH.)

In this context, we synthesize research advances in 2D material-based novel artificial neurons and synapses, covering biomimetic models, physical mechanisms, performance metrics, reconfigurable strategies, and their applications, as shown in Fig. 2. At the device level, special emphasis is given to the conductance-dependent and conductance-independent biomimetic models, coupled with the intrinsic volatile switching mechanisms observed in artificial neuron devices fabricated from 2D materials. Subsequently, various underlying physical mechanisms governing synapse behavior are systematically analyzed, alongside an examination of performance metrics in advanced synaptic devices. A comprehensive categorization of reconfiguration strategies in neurons and synaptic devices highlights three dominant switching methods: terminal programming, input parameter regulation, and materials property modulation. At the system level, the integration and application of artificial neuromorphic devices across multimodal sensing, pattern recognition, and logical operation are also demonstrated. Furthermore, we critically analyze persistent challenges in neuromorphic devices and integrated systems, while providing a forward-looking roadmap on their transformative potential in brain-like intelligent chips.

The structure of this article is divided into the following parts: The introduction outlines the fundamental limitations of current computing paradigms at the hardware level and articulates the necessity of pursuing neuromorphic engineering based on 2D materials. Section 2 outlines the biological inspiration for artificial nervous systems, along with the corresponding hardware-level correlations and the behavioral characteristics of such systems. Section 3 provides a detailed summary of bio-inspired models and physical mechanisms of artificial neurons. Section 4 analyzes performance metrics for artificial synaptic devices, elucidating the underlying physical mechanisms governing their operation. Section 5 advances the discussion to reconfiguration methodologies that enable adaptivity and scalability in neuromorphic devices. Various implementations of neuromorphic devices in integrated interconnect, perception, recognition, and logic operations are presented in Sect. 6, followed by an analysis of critical hardware challenges and a roadmap for brain-like chips in intelligent systems in Sect. 7.

## Bio-inspired Artificial Nervous System

**Fig. 3:**
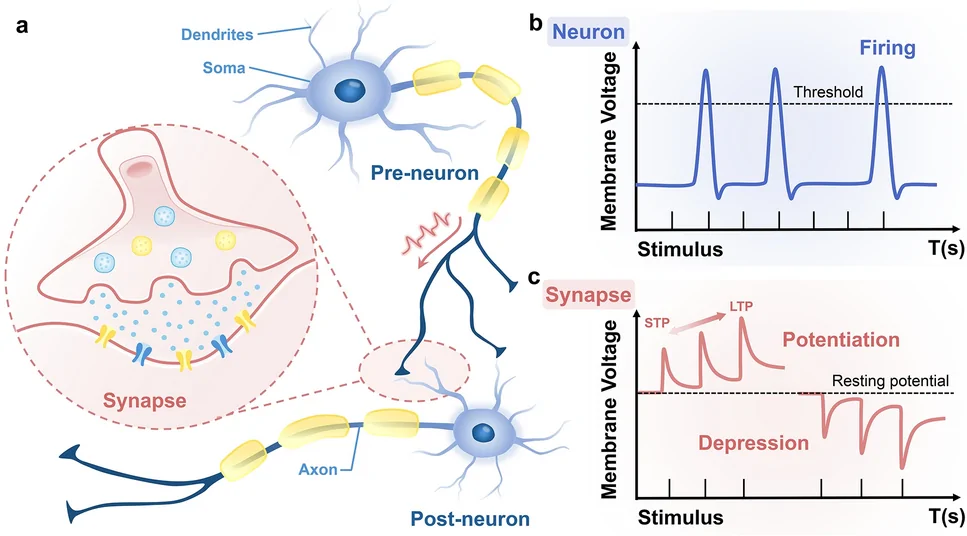
Typical signal transmission diagram in living organisms. **a** Schematic diagram of information transmission between the pre-neuron and post-neuron. **b** Diagram of changes in the membrane potential of neurons induced by input signals. **c** Schematic diagram of postsynaptic membrane potential changes in response to excitatory and inhibitory stimuli

The central nervous system of living organisms is a highly sophisticated regulatory network, with neurons serving as its fundamental structural and functional units. Figure 3a illustrates the typical signal transmission structure, which primarily consists of the soma, neurites (dendrites and axons), and synapses. The soma is responsible for integrating all incoming signals and making the final decision. The dendrites and axons within neuronal processes are specialized for signal reception and transmission, respectively. Synapses serve as functional connections that mediate communication between neurons or between neurons and sensory/effector cells. Information transmission within living organisms mainly includes the following stages:

- Resting state. In the absence of stimulation, neurons sustain a dynamic equilibrium characterized by selective membrane permeability and concentration gradients of ionic (e.g., K⁺, Na⁺) across the membrane, thereby establishing the resting potential.
- Depolarization. When neurotransmitters activate the receptors on the dendrites, the membrane’s selective permeability alters, triggering a Na⁺ influx that elevates the membrane potential.
- Repolarization. The neuronal soma undergoes spatiotemporal summation of excitatory and inhibitory synaptic inputs. Upon reaching the threshold potential, action potentials are initiated at the axon initial segment. Temporally sequential action potentials constitute spike trains through frequency and interval encoded information features, such as internal pressure, visual edges, and sound frequency.
- Refractory period. Following the initiation of action potentials, Na⁺ channels rapidly inactivate, inducing a refractory period that persists until resting membrane characteristics are restored. The resulting spike propagates unidirectionally along the axon in an all-or-none manner with no amplitude decay.
- Synapse transmission. The arrival of spike trains at synaptic terminals initiates a cascade of electrochemical events that mediate interneuronal communication. The efficiency of interneuronal information transfer is directly governed by the strength of synaptic connections, which is dynamically regulated through synaptic plasticity. High-frequency and repeated neural activities drive the transformation of synaptic plasticity from short term (short-term plasticity, STP) to long term (long-term plasticity, LTP), serving as the core mechanism for learning and memory in the brain.

Artificial neural systems aim to construct large-scale distributed processing integrated circuit architectures by imitating the structure and function of biological neural systems. Such system comprises integrated networks of neuromorphic devices, where the two fundamental components, artificial neurons and artificial synapses, have a direct hardware mapping relationship with biological counterparts: the former simulates the integration and firing function of biological neurons, converting the electrical signal of membrane potential changes (input) into a discrete spike train output (Fig. 3b); the latter replicate the signal transmission and plasticity of biological synapses, with their conductance states directly corresponding to synaptic weights (Fig. 3c). The operation of artificial neural systems adheres to an event-driven processing logic. Spatiotemporally encoded external inputs drive neurons to generate sparse spike trains. As these spike trains are transmitted to the synaptic array, they modulate synaptic weights accordance with learning rules, enabling spatiotemporal correlation learning and feature extraction. Based on this process, artificial neural systems can reproduce the core information processing mode of biological neural systems at the hardware level, providing critical support for the breakthrough of brain-inspired computing.

## 2D Materials-based Dedicated Artificial Neurons

As mentioned above, biological neurons function as spatiotemporal integrators through synaptic weighting. Such characteristic has been abstracted into various mathematical models and hardware implementations. The further demand for model simplification is driving the exploration of neuron devices with threshold switching (TS) and volatile dynamic characteristics in 2D material platforms to achieve more integrated and intelligent neuromorphic hardware systems.

### Neuron Models

**Fig. 4:**
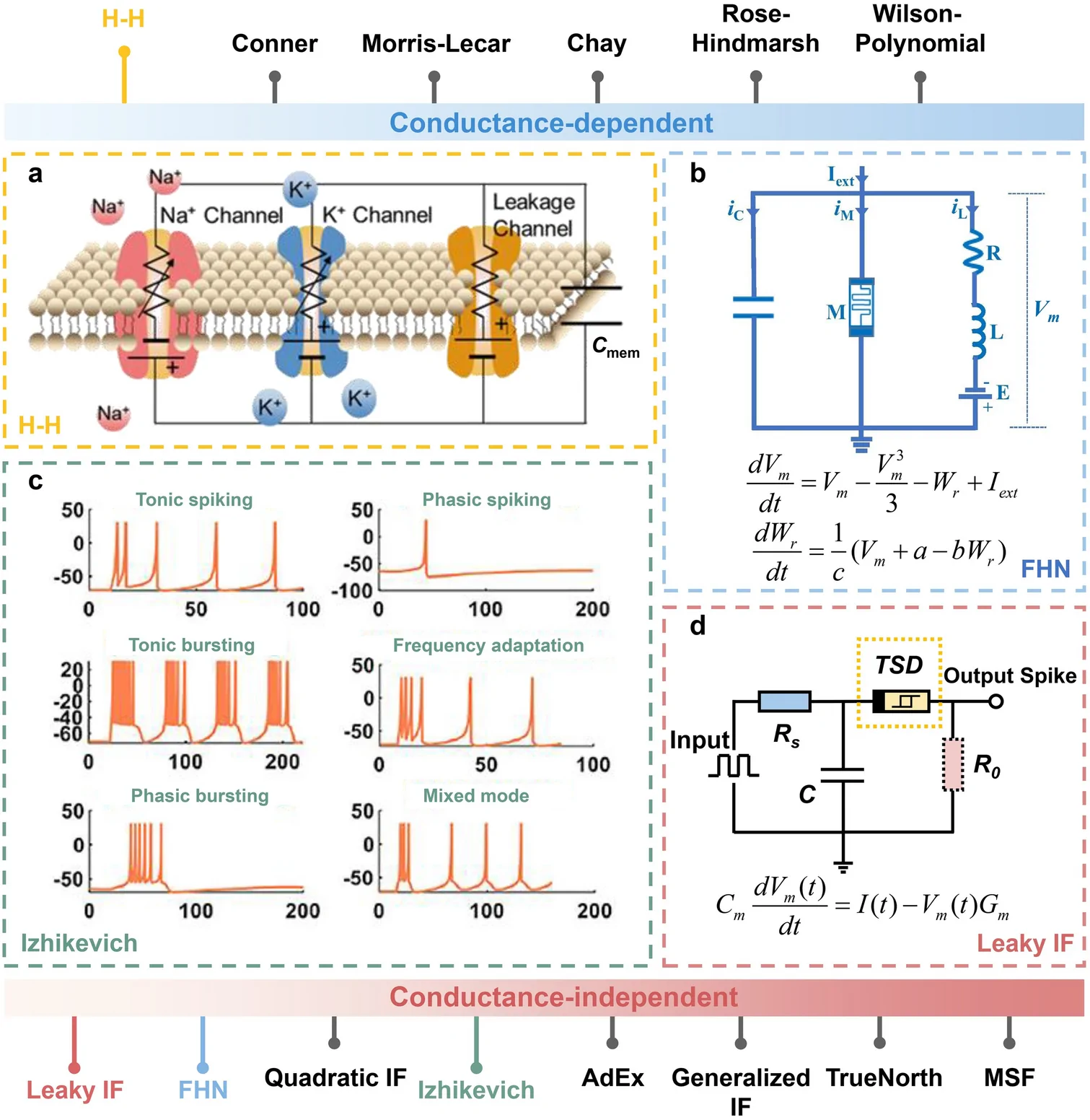
Various neuron models and classic spiking patterns. **a** Equivalent circuit of the H–H neuron model. The dynamics of Na^+^ and K^+^ channels on the cell membrane regulate the generation of action potentials**.** Reproduced with permission [20]. Copyright (2024), Wiley–VCH GmbH. **b** Diagram of the FHN neuron circuit and corresponding ODEs (*V*_m_ and *W*_r_ are the membrane potential of the neuron and the recovery variable. *I*_ext_ is the intensity of the externally applied current. *a*, *b*, and *c* are three constants). Reproduced with permission [22]. Copyright (2023), Elsevier. **c** Six classic spiking patterns are generated by the Izhikevich model. Reproduced with permission [23]. Copyright (2022), Frontiers Media S. A. **d** A typical RC-based LIF neuron circuit with general TSDs, and the corresponding ODE (*V*_m_(t) and *I*(t) represent the membrane potential and the total input current at time *t*. *G*_m_ and *C*_m_ are the membrane conductance and membrane capacitance of the neuron)

Neuronal models can be categorized into two primary types: conductance-dependent and conductance-independent. Conductance-dependent models simulate neuronal behavior by replicating the specific electrophysiological properties of the neuronal cell membrane to reconstruct its conductive channels. The Hodgkin–Huxley (H–H) neuron is a classic conductance-dependent model that formulates a set of differential equations describing the membrane potential and ion-channel gating variables, thereby establishing the electrophysiological modeling of neuronal ionic dynamics. The physical implementation of mathematical descriptions is achieved through equivalent resistance–capacitance (RC) circuit methodology [20]. In this circuit, the phospholipid bilayer membrane and transmembrane ion concentration gradients were represented as a capacitive element and electromotive force sources, respectively. K^+^/Na^+^ channels were characterized as variable conductance elements, while other leak channels are equivalent to a linear conductance (as illustrated in Fig. 4a). This model establishes a quantitative framework for understanding action potential generation through precise biophysical components, where the nonlinear interactions of these electrical elements faithfully reproduce multiple spiking activities observed in biological neurons. Subsequently, a series of more simplified conductance-dependent neuronal models capable of simulating a wider range of firing patterns were developed (refer to the upper axis in Fig. 4). These neuron models are specifically designed to capture the unique electrophysiological characteristics of different neurons. For instance, the Morris–Lecar neuron model emphasizes the conductivity of membranes and the types of neuron ion channels, while Chay describes bursting patterns and chaotic behavior in neurons.

However, the excessive number of dynamic variables of these models results in inefficient computation for large-scale networks. The conductance-independent neuron models have been further explored in depth (refer to the below axis in Fig. 4). It aims to describe the input–output behavior of neurons through abstract mathematics, focusing on a functional perspective rather than on the specific physiological structure. Therefore, a FitzHugh–Nagumo (FHN) neuron model with simplified dynamic variables was developed (Fig. 4b) [21, 22], which captures the essential dynamics of neuronal excitability, including the accommodation and anode break excitation characteristics. In addition, the Izhikevich neuron model requires only two ordinary differential equations (ODEs) for membrane potential and recovery variable, along with discrete reset rules, to reproduce over 20 firing patterns, including tonic spiking, tonic bursting, and phase spiking, among others (Fig. 4c) [23]. This model achieves a computational cost two orders of magnitude lower than that of H–H-type models [24, 25], striking a balance between computational efficiency and biological plausibility. Another classic simplified structure capturing the general process of neural spike transformation is the leaky integrate-and-fire (LIF) neuron model. It focuses on the integration dynamics of the membrane potential: the potential accumulates and rises with the arrival of input pulses, while spontaneously decaying due to leakage. Once it crosses the firing threshold, a spike is triggered, and the integrated state is immediately reset. Although the LIF model lacks the rich and intricate behaviors of biological neurons, it retains the two core functions of “integration” and “threshold-triggering”. Consequently, it has been widely adopted for large-scale circuit simulations and real-time spike signal processing. The hardware implementation of the LIF model typically utilizes a threshold switching device (TSD) (or specific circuit block) coupled with a charge-storing capacitor and a leak resistor. As shown in Fig. 4d, when an input voltage pulse is applied, the capacitor initiates charge accumulation. Once the capacitor voltage exceeds the threshold (*V*_th_), the TSD switch abruptly from a high-resistance state (HRS) to a low-resistance state (LRS). As a result, the artificial neuron generates a spike and discharges the capacitor through the TSDs (reset). When the voltage across the TSD drops below the holding voltage (*V*_hold_), it returns to the HRS and enters a refractory period [26, 27], completing a full leaky integrate-and-fire cycle, which can be repeated with continuous input while maintaining consistent firing amplitude regardless of pulse accumulation [28]. The LIF equivalent circuit and corresponding ODE are shown in Fig. 4d. In LIF models, when the input pulse interval significantly exceeds the RC circuit’s decay time constant (*T*_pulse_ ≫ *τ* = RC), the capacitor fully discharges (leaky) through the resistor *R*_s_ to attain the resting potential prior to the arrival of subsequent pulses, preventing sufficient charge accumulation to attain the threshold voltage, thereby inhibiting spike generation. During short pulse intervals, the capacitor exhibits minimal leakage, which facilitates near-ideal charge accumulation mimicking integrate-and-fire (IF) behavior. In large-scale neural network implementations, omitting the leak resistor from LIF hardware models remains capable of capturing key neuronal characteristics, significantly reducing computational complexity and memory requirements [29]. Furthermore, neuron models have evolved to incorporate more physiologically meaningful and computationally efficient conductance-independent models, such as quadratic IF and multi-synaptic firing (MSF).

With the progress in neuronal hardware implementation, neuron dynamics of the H–H and LIF models have been effectively realized through memristive systems [30, 31]. More importantly, individual memristive devices with volatility and TS capabilities could also emulate certain classical neuronal behaviors by the progression of physical or chemical processes that drive electrical switching. These devices eliminate the need for complex external circuitry and hold significant potential in area efficiency, thereby propelling the development of artificial neural systems from mathematical models toward the construction of large-scale hardware systems.

### Mechanisms of Artificial Neuron Devices

2D materials are excellent platforms for constructing memristive neuron devices and exhibit unique advantages in simulating high energy efficiency and complex neural dynamics due to their intrinsic property. The in-depth exploration and precise control of the intrinsic physical mechanisms of memristive devices is a focus of current research, which can primarily be categorized into the following five mechanisms:

#### Ion Migration Neurons

**Fig. 5:**
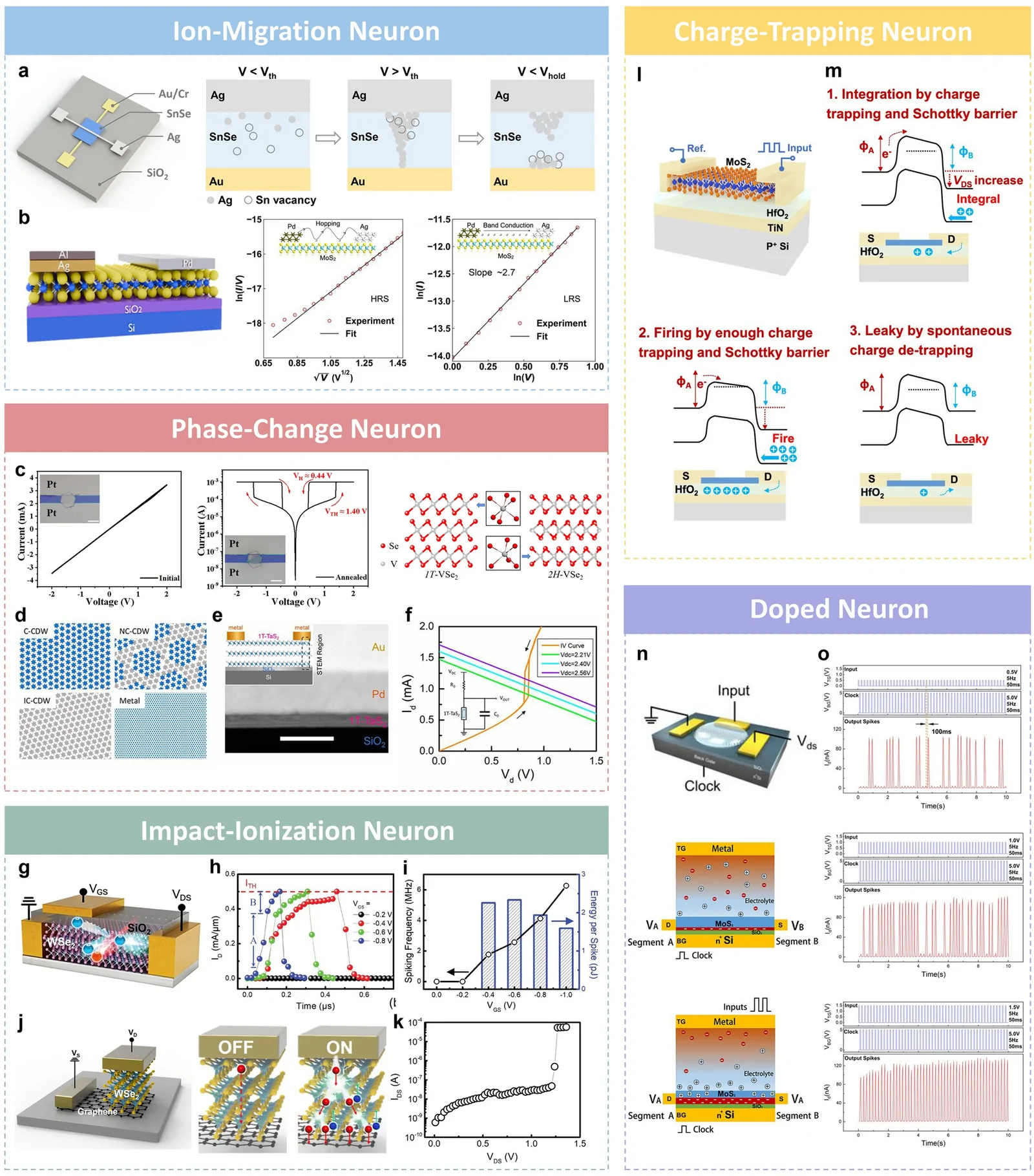
Artificial neuron mechanisms and electrical characteristics. **a**, **b** Ion-migration neurons: **a** Schematic illustration of the Ag/SnSe/Au device and the TS mechanism. **b** Cross section schematic of the MoS_2_-based TS device and high- and low-resistance switching mechanisms. Reproduced with permission [43]. Copyright (2024), Wiley–VCH GmbH. **c**, **d** Phase-change neurons: **c** Electrical measurement results of fabricated Pt/VSe_2_/Pt memristors before and after annealing, with corresponding phase transition schematics. Reproduced with permission [47]. Copyright (2024), The Royal Society of Chemistry. **d** Atomic structure of the four phases of 1 T-TaS_2_. **e** Cross section and schematic structure of the 1 T-TaS_2_ oscillator. **f** *I-V* characteristics of the 1 T-TaS_2_ device under different bias conditions. Reproduced with permission [48]. Copyright (2021), American Chemical Society. **g**, **k** Impact-ionization neuron: **g** Schematic representation of the 2D WSe_2_ impact ionization device. **h** Transient current characteristics of the channel under fixed source–drain bias with varying gate voltages. **i** Spiking frequency and energy consumption of the device with varying gate voltages. Reproduced with permission [49]. Copyright (2025), Wiley–VCH GmbH. **j** Schematic of WSe_2_/graphene heterostructure impact ionization device and the TS mechanism. **k** A sharp increase in current due to impact ionization. Reproduced with permission [50]. Copyright (2023), The Royal Society of Chemistry. **l**, **m** Charge-trapping neurons: **l** Structure of the MoS_2_-based neuron. **m** Energy band diagrams illustrating the working mechanism corresponding to the LIF process. Reproduced with permission [15]. Copyright (2022), American Chemical Society. **n**, **o** Doped neuron: **n** Schematic illustration of the MoS_2_ neuristor and the TS mechanism.** o** Integrate-and-fire function of the MoS_2_ neuristor under stimulation by input and clock signals. Reproduced with permission [52]. Copyright (2019), American Chemical Society

Ion migration dynamics represent one of the most widely utilized mechanisms in current 2D material-based neuron devices, which encompasses two categories: metal ion migration and vacancy migration. In metal–semiconductor–metal (MSM) structures, active electrodes (e.g., Ag, Cu) generate metal ions by an electrical potential (M → M^n+^ + ne^−^). Subsequently, ions migrate through the van der Waals gaps [32, 33] or surfaces [34] of 2D functional layer before being progressively reduced to form ultrathin conductive filaments connecting the two electrodes, triggering a current spike. The potential leakage may originate from the thermal diffusion of ions. Upon removal of the electric field, these ultrathin conductive filaments spontaneously dissipate, corresponding to the reset phase. This process is defined as electrochemical metallization (ECM) [35], which is governed by electric field strength, temperature, and material defects. Alternatively, intrinsic vacancies in the function layer are driven to migrate directionally by the electric field or thermal excitation. Functioning as charge traps or ion transport pathways, these vacancies dynamically modulate local conductivity. This vacancy-dominated TS mechanism is referred to as the valence change mechanism (VCM) [36, 37]. In practice, intrinsic kinetic coupling exists between metal ions and vacancies. The provision of diffusion pathways for metal ions by intrinsic defects in 2D materials (e.g., vacancies, grain boundaries) significantly lowers the activation energy for migration, directly resulting in faster switching speeds and near-biological energy efficiency [38–41]. Building on this kinetic correlation, Qin et al*.* enabled the controlled formation of Ag conductive filaments in SnSe (Fig. 5a) [42]. The quantity and spatial position of Ag conductive filaments are modulated by the dynamic distribution of Sn vacancies, generating stochastic threshold voltages that closely emulate the flexibility of biological neuronal firing. In addition, the formation and rupture locations of conductive filaments vary depending on the local electrical conductivity, defect distribution, and interface properties of 2D materials.

Moreover, Cruces et al*.* developed a lateral MoS_2_ device (Fig. 5b) [43] utilizing a distinct mechanism that differs from continuous filament formation. This design achieves repeatable volatile resistance switching through controlled Ag^+^ surface migration across multilayer MoS_2_. When the device is initially in the HRS, low-concentration extended clusters of Ag nanoparticles or Ag_2_S distribute across the MoS_2_ surface, exhibiting Poole–Frenkel hopping conduction. Applied bias increases Ag concentration, thereby modifying the band structure of MoS_2_ to shift conduction from localized state hopping to a space-charge-limited conduction mechanism, resulting in a transition to the LRS with significantly enhanced current.

#### Phase-Change Neurons

Certain 2D materials exhibit reversible phase transitions in response to external stimuli, closely emulating the stimulus-firing behavior of biological neurons. Specifically, when input signals exceed the phase-change critical threshold, the material undergoes a rapid phase transition, inducing abrupt changes in conductivity that emulate neuronal action potential firing. This process typically involves coupled electronic and structural phase transitions through energy accumulation. These structural transitions primarily involve lattice rearrangement and symmetry breaking, while electronic phase transitions entail significant band structure reconstruction and altered electronic correlation effects [44–46]. As shown in Fig. 5c, Zhong et al*.* demonstrated that annealed VSe_2_ undergoes atomic rearrangement to form a 2H phase with AB stacking sequence [47]. Voltage-induced Joule heating then converts it to a 1T phase with AA stacking, inducing TS behavior in the device. In addition, Liu et al*.* reported that the neuronal oscillation mechanism involves electric-field-driven switching between nearly commensurate and incommensurate phases in 1T-TaS_2_ films. This system exhibits biologically realistic stochastic firing behavior originating from melt-quench-induced reconfiguration of charge density wave domains (Fig. 5d–f) [48]. The reset phase in phase-change neurons occurs through thermal dissipation mechanisms that gradually restore the initial state.

#### Impact Ionization Neurons

Impact ionization neurons represent another class of devices based on the semiconductor avalanche effect. Under a strong electric field, charge carriers are accelerated and gain sufficient kinetic energy, which leads to collision ionization with lattice atoms and triggers an avalanche multiplication of carrier concentration. This process enables energy-efficient spike generation in a short transient period, thereby simulating the integration-fire behavior of biological neurons. Lee’s team demonstrated an impact ionization field-effect transistor neuron using WSe_2_ as the channel material (Fig. 5g–i) [49]. When the applied bias (*V*_gs_ = − 0.4 V, *V*_ds_ = 2 V) exceeds the critical field strength in ungated regions, carrier kinetic energy surpasses the impact ionization threshold, triggering an exponential current surge (Phase A) that emulates action potential firing. As carrier density increases in the channel, enhanced carrier–carrier scattering and stochastic collisions cause more energy loss, requiring a higher bias voltage to sustain impact ionization. This results in a more gradual increase in current during Phase B. Upon withdrawing the gate electric field, carrier depletion enables automatic reset to the initial state. This reliable and repeatable LIF behavior demonstrates functional spiking neuron operation. Notably, this neuron device exhibits spiking behavior at a low critical field, a 565 ns transient response, and an energy consumption of approximately 2 pJ per spike, all enabled by the high impact ionization coefficient of WSe_2_. Similar impact ionization TS was reported in their other work using a two-terminal vertically stacked WSe_2_/graphene heterostructure [50]. When the bias exceeds the avalanche breakdown voltage (~ 1.2 V), an abrupt current surge occurs via carrier multiplication (Fig. 5j, k). However, these neuronal devices require further optimization, such as precise electric field control to prevent irreversible breakdown, moderate bandgap engineering to balance impact ionization efficiency and leakage current, as well as fatigue resistance and thermal management for stable high-frequency pulsed operation.

#### Charge-Trapping Neurons

The charge-trapping and detrapping processes can effectively emulate neuronal integration, firing and leaking. As demonstrated by Huo et al*.*, a quasi-volatile MoS_2_ neuron utilizing charge trapping and Schottky barrier modulation is shown in Fig. 5l, m [15]. Charge carriers injected from the terminal electrodes become trapped at defects in the dielectric layer, driving neuronal integration. When the accumulated charge reaches the transistor’s threshold voltage, channel carriers are abruptly triggered, generating a spiking pulse. Upon voltage removal, charges trapped in shallow-level defects spontaneously de-trap, governing the reset process. Additionally, Wang et al*.* proposed an alternative approach using source–drain voltage to manipulate carrier tunneling into floating-gate layers for channel activation [51]. This configuration establishes a positive feedback loop between source-injected current, impact ionization, and floating-gate potential, significantly enhancing tunneling efficiency to produce abrupt TS behavior.

#### Doped Neurons

A doping-based neuron operates by introducing charges or ions into the resistive switching layer through external stimuli, resulting in a transient change of state. Bao et al*.* demonstrated a method where Li⁺ in the top gate electrolyte migrates under applied voltage, inducing reversible electrochemical doping of the channel layer that dynamically modulates its threshold voltage. The bottom-gate clock signals then regulate drain current spiking through field-effect control, as illustrated in Fig. 5n, o [52].

In practice, the information integrated by artificial neurons is encoded in output signal amplitude, frequency, waveform characteristics, among others. These output characteristics are modulated by the input signals, synaptic activity, and the neuronal intrinsic plasticity. Among them, neuronal intrinsic plasticity is achieved through amplification of excitatory postsynaptic potentials, adjustment of spike threshold, and alteration of resting membrane potential. Some initial hardware implementations of neural intrinsic plasticity have already been explored. An IF neuron module based on wafer-scale monolayer MoS_2_ films that can adaptively regulate the neuronal membrane resting potential for time-to-first-spike encoding by emulating the intrinsic plasticity of neurons has been developed by Zhou’s group [53]. Moreover, several studies have replicated adaptive threshold regulation ability through approaches such as external circuit control [54], functional material doping [52], defect engineering [55, 56], and modality control [57, 58]. For instance, a threshold-type memristor utilizing the 2D V_2_C/V_2_O_5−x_ heterojunction has been demonstrated [58]. The device exhibits a threshold voltage that can be linearly tuned by the power density and the wavelength of near-infrared light, a feature enabled by the strong NIR absorption of V_2_C and the volatile switching induced by oxygen vacancies in V_2_O_5−x_. Lee et al*.* showed electric-field-driven Ag⁺ migration that dynamically modulates trap-state density in GeSe_2_ channels, enabling continuous threshold voltage adjustment [55]. Overall, artificial neurons based on different device architectures and materials exhibit distinct response mechanisms. Neuronal excitability and signal integration efficiency can also be regulated through intrinsic plasticity, thereby optimizing input–output relationships. For a more detailed comparative analysis, Table 1 summarizes the operating principles and performances of the reported 2D material-based neuronal devices.

**Table 1:** Mechanisms and performances of artificial neuron devices based on 2D materials

Materials	Construction	Mechanism	Neuron model	Threshold (V)	On/off ratio	Energy or power consumption (per spike)	Multimodal (yes/no)	References
ITO/2D TiO_x_/Au	1M^*a*^	/	LIF	− 1.9 to − 2.2	10^9^	5 nJ	N	[59]
Ag/MoS_2_/TiW	1M	ECM	LIF	0.9 to1.5	~ 10^5^	1.4 μW	N	[60]
Ag/Ag (NPs)/MXene/ITO	1M	ECM	IF	~ 0.93	10^3^	/	Y	[61]
Ag/O-MXene/SiO_2_/Si	1M	ECM/VCM	IF	2.8	3 × 10^4^	Without/With light: 74 μJ/25 μJ	Y	[62]
Cu/MXene/Cu	1M	ECM	LIF	~ 0.68	/	20 nJ	N	[63]
Ag/MoS_2_/Au	1M + 1C^*b*^2R^*c*^	ECM	LIF	~ 0.35 to 0.4	10^6^	/	N	[64]
Al/Ag/MoS_2_/Pd	1M	ECM	LIF	~ 2.1	/	/	N	[43]
Ag/SnSe/Au	1M	ECM	LIF	< 0.6	~ 10^4^	/	N	[42]
Au/Ag/Al_2_O_3_/Gra/MoS_2_/SiO_2_	1M	ECM	LIF	~ 0.17	~ 10^6^	/	N	[65]
Ag/Ti/GaSe/Pt/Ti	1M + 1C2R	ECM/VCM	LIF	~ 0.3 to 0.42 (without Ar plasma) ~ 0.4–0.75 (with Ar plasma)	~ 10^6^ ~ 10^5^	/	N	[66]
Cu/SnS_2_/Cu	1M	ECM/VCM	IF	0.3	94	/	N	[56]
Ag/Ti/HfSe_2-x_O_y_/Pt	1M + 1C1R + feedback circuits	ECM and Interfacial Engineering	LIF	0.42–0.65	~ 10^6^	/	N	[67]
Ni/Graphene/v-MoS_2_/Ni	1M + 1C3R	Ion Migration	IF	2.9–4	> 10^2^	8 μW	N	[68]
Au/CuInP_2_S_6_/Cu	1M	Ion Migration	LIF	~ 0.82	10^7^	/	N	[69]
Cu/CuInP_2_S_6_/Graphene	1M	Ion Migration	/	~ 0.8	104	/	N	[14]
Ag/MXene (V_2_C)/W	1M	ECM/Joule Heat Effect	LIF	~ 3.1	/	/	N	[70]
Ag/MXene/GST/Pt	1M	ECM	IF	0.38	> 10^3^	/	N	[71]
Au/Pd/1 T-TaS_2_/SiO_2_	1M + 1C1R	Phase Transition	Oscillation	0.823–0.84	/	/	N	[48]
Pt/VSe_2_/Pt	1M	Phase Transition	LIF	~ 1.46/ ~ − 1.5	> 10	/	N	[47]
Au/WSe_2_/Au Au (TG)	1M	Impact Ionization	LIF	*V*_gs_ = − 0.37 (@*V*_ds_ = 2)	/	2 pJ	N	[49]
Au/Graphene/WSe_2_/Au	1M	Impact Ionization	/	~ 1.2	/	/	N	[50]
Au/MoS_2_/Au HfO_x_ (dielectric) TiN (BG)	1M	Charge trapping	LIF	*V*_ds_ > ± 3	10^3^	/	N	[15]
Drain/ZnPc-modified MoS_2_/Source graphene (FG) Si (BG)	1M	Charge trapping and impact ionization	IF	*V*_ds_ = 6.5	10^8^	/	N	[51]
Au(D)/MoS_2_/Au(S) PEO:LiClO_4_ (TG) n^+^Si (BG)	1M	Electrochemical Doping	IF	*V*_gs_ = 0.9 to 1.2	/	/	N	[52]

^*a*^*M* Memristive device, ^*b*^*C* Capacitor, ^*c*^*R* Resistor, ^*d*^*V*_ds_ Drain–source voltage, ^*e*^*V*_gs_ Gate–source voltage, ^*f*^*TG* Top gate, ^*g*^*BG* Bottom gate, ^*h*^*FG* Float gate

## 2D Materials-based Dedicated Artificial Synapses

As critical components in neural networks, artificial synaptic handle the core functions of information transmission, processing, and memory. Leveraging 2D materials as a frontier platform, related studies are promoting the precise emulation of biological synaptic behavior through the key path of mechanism innovation and performance optimization. This involves unraveling novel mechanisms at the atomic scale while building upon established ones to develop more versatile, high-performance, and highly energy-efficient synaptic hardware.

### Mechanisms of Artificial Synapse Devices

The core attributes of artificial synaptic devices lie in their non-volatile memory and continuous conductance modulation, enabling data processing and computation. To date, researchers have elucidated several key mechanisms based on the performance and characterization of 2D material-based synapses:

#### Conductive Filament Synapses

**Fig. 6:**
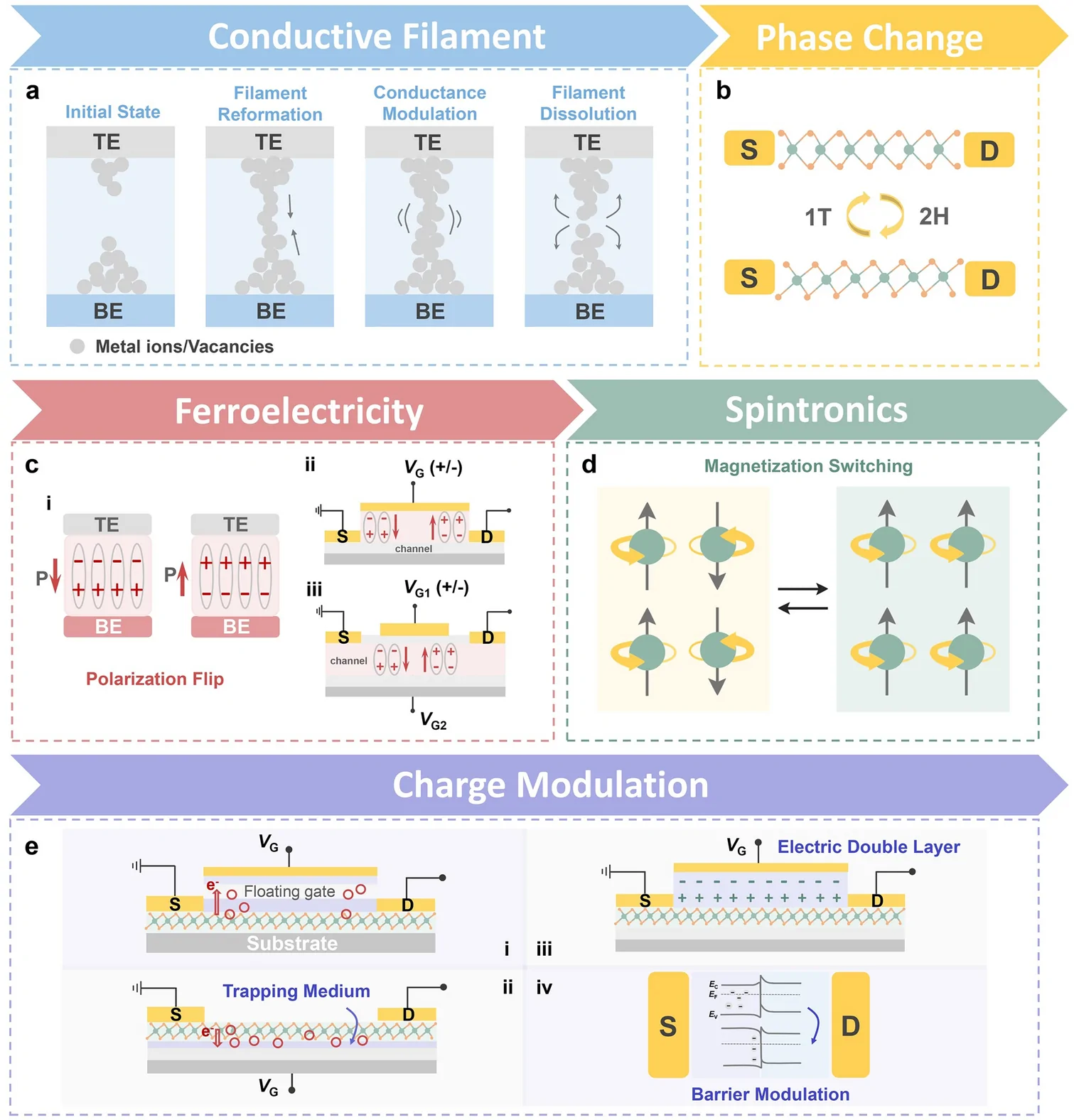
Schematic illustrations of intrinsic mechanisms in various artificial synaptic devices. **a** Conductive filament synapses. Electric-field-driven Conductive filament gradual formation and dissolution via active metal ion or vacancy migration enables synaptic plasticity emulation. **b** Phase-change synapses. Reversible interphase transitions induce conductance modulation in artificial synapses. **c** Ferroelectric synapses. Synaptic weight modulation through reversible ferroelectric polarization switching. **d** Spintronic synapse. The magnetic state of the magnetic layer is typically modulated by STT or SOT. **e** Charge modulation synapses. The biological synaptic behavior is achieved by controlling charge distribution, storage, and release states

This mechanism of artificial synapse also follows ECM/VCM, mentioned earlier, controlling the resistance state through the formation of conductive pathways [72–75]. Although artificial synapses and neurons share fundamental operating mechanisms, their distinct functional specifications lead to divergent filament dynamics. In contrast to the transient filament formation in neuronal devices, the conductive filaments in synaptic devices exhibit retention and gradual dissolution upon withdrawal of the electrical stimulus, attributable to characteristics of the input signals, the dielectric material, or the filament composition. This non-volatile characteristic enables continuous modulation of the synaptic conductance, which is controlled by the quantity [76], thickness [16], and spatial distribution [77] of the conductive pathways bridging the electrodes, as illustrated in Fig. 6a. Additionally, functional buffer layers are often employed to optimize gradual synaptic switching behavior [78, 79]. The effectiveness of this approach is demonstrated in a novel GO/Py-salt/GO trilayer memristor for controlled conductive filament ordering. The uniform Py-salt interlayer effectively serves as a buffering interlayer to regulate metal ion migration and filament growth, enabling progressive conductance modulation and stable bidirectional tuning [79].

#### Phase Change Synapses

The utilization of phase transition properties in 2D materials provides effective strategies for emulating synaptic functions. In widely investigated layered phase-change materials such as MoTe_2_ and MoS_2_, phase transitions between semiconductor and metallic phase [80], or between crystalline and amorphous states [81], can be controllably induced by various stimuli including irradiations [82, 83], electric fields [84, 85], doping [86, 87], pressure [88], and thermal activation [89, 90] (Fig. 6b). Such non-volatile progressive modulation of conductance states is attributed to the appropriate growth and shrinkage of the crystalline phase [91], thereby facilitating synaptic weight update and memory retention. Although relying on a similar phase-change mechanism, the slow, cumulative response of synaptic devices stands in contrast to the transient dynamics of neuronal elements. This functional differentiation depends on the intrinsic purity of the material and the stability of its metastable states [44, 92]. Furthermore, leveraging such tunability provides a design strategy for achieving reconfigurable neuromorphic functionalities within a unified material system.

#### Ferroelectric Synapses

2D ferroelectric materials with asymmetric structures exhibit macroscopic spontaneous polarization [93]. The polarization can be switched by an external electric field while retaining the altered state. Such ferroelectric polarization is widely utilized in two-terminal and three-terminal memristive devices, as shown in Fig. 6c. Wang et al*.* fabricated a second-order memristor based on 2D SnSe thin films, where ferroelectric polarization of SnSe modulates the interfacial barrier at the SnSe/NSTO junction to emulate biological synaptic properties (Fig. 6c(i)) [94]. In the ferroelectric synaptic transistor, ferroelectric materials are often used as the gate dielectric, where only polarization-bound charges exist, and polarization switching is employed to modulate the channel conductance (Fig. 6c(ii)) [95]. Additionally, transistors utilizing the 2D ferroelectric semiconductor α-In_2_Se_3_ as channel material have also been demonstrated to effectively emulate synaptic plasticity. This functionality relies on the controllable and cumulative effects of ferroelectric polarization, enabled by direct control of channel polarization switching through the gate (Fig. 6c(iii)) [96].

#### Spintronic Synapses

The nonlinear and non-volatile spin dynamics fundamentally enable magneto-resistive devices to exhibit synaptic behaviors. Typically, the magnetic states (magnetization direction or magnetic domains) of the magnetic layer can be modulated by spin-transfer torque (STT) or spin–orbit torque (SOT). The alteration in magnetic states is transformed into changes in resistance through the magnetoresistance effect, thereby emulating the plasticity of synaptic weights (as shown in Fig. 6d). Consequently, a series of spin-electronic synaptic devices based on 2D materials with relatively high Curie temperatures and strong perpendicular magnetic anisotropy have been developed. Representative work by Yang et al*.*, device resistance correlates pronouncedly with magnetic domain wall numbers, decreasing as walls are eliminated. Current-driven manipulation of domain wall density in Fe_3_GeTe_2_ enables reconfigurable spin structures and multi-state resistive switching. The device further supports reversible resetting of both domain walls and resistance through high-amplitude pulse-induced thermal demagnetization, thereby reproducing biological synaptic strengthening and weakening processes [97]. Moreover, by exploiting SOT-driven magnetization switching in a Bi_2_Te_3_/CrTe_2_ heterostructure, Huang et al. also achieved multi-state tuning of the Hall resistance, demonstrating highly linear and symmetric long-term potentiation and depression [98].

#### Charge Modulation Synapses

Charge modulation synapses operate through the injection, storage, and release of charge. External stimuli control this process to alter the barrier heights or carrier concentrations within the device, thereby modulating the continuous conductance of the synaptic device. Several charge-modulation approaches have been extensively explored for synaptic devices, including employing floating gates, charge-trapping layers, electric-double-layer structures, and film interfaces. In floating-gate transistors, electrons tunnel from the channel into the floating gate and become trapped (Fig. 6e(i)). The stored charge modulates channel conductivity, thereby emulating synaptic weight plasticity [99]. Given the availability of 2D materials spanning from metals to insulators, all-2D floating-gate synaptic devices can be designed and fabricated utilizing metallic materials like graphene as the floating gate, insulating materials like h-BN as the tunneling layer, and semiconducting materials as the channel layer [100, 101]. Several studies have achieved modulation of channel carrier concentration by designing charge-trapping layers (Fig. 6e(ii)) [102–104]. For instance, in a MoS_2_/GaPS_4_ heterojunction transistor, MoS_2_ serves as the readout layer while GaPS_4_ functions as the charge-trapping layer [102]. When a positive gate pulse is applied, electrons from MoS_2_ overcome the interface barrier and are trapped in defect states within the GaPS_4_ layer. Upon removal of the positive gate pulse, these electrons remain confined in the defect states of the GaPS_4_ layer. The trapped electrons generate a negative electric field in the MoS_2_ channel, thereby reducing the channel current. Conversely, a negative gate pulse results in a continuous increase in the channel current. Moreover, the realization of an electric-double-layer (EDL) synaptic transistor relies on the migration and rearrangement of ions within an electrolyte under the gate bias. When a gate voltage is applied, ions in the electrolyte migrate directionally to form an EDL at the channel/electrolyte interface, a process analogous to the diffusion of neurotransmitters across a synaptic cleft [105]. Such EDL induces a strong electrostatic doping effect in the channel, thereby modulating its conductivity [106, 107] (Fig. 6e(iii)). In addition to electrical control, optically induced ion-gating effects have also been validated. For instance, in the approach proposed by Jeong et al*.*, the photogating effect on MoS_2_ induces ion migration within the dynamic cation reservoir provided by the SA layer, thereby modulating the persistent photoconductivity (PPC) behavior. This ion-mediated PPC can simulate synaptic plasticity that responds to the 680 nm illumination [108]. Another approach to modulating charge for synaptic behavior involves controlling the charge distribution at the interface, thereby altering the energy barrier (Fig. 6e(iv)). For instance, in a MoSe_2_/MoS_2_ heterojunction-based memristor, a type-II heterojunction naturally forms due to the difference in their Fermi levels. A high energy barrier exists at the heterojunction interface, hindering the free movement of electrons. When a positive voltage is applied, S^2−^ gradually accumulates at the MoSe_2_/MoS_2_ interface. This charge accumulation induces band bending at the heterojunction, effectively reducing the height of the interface barrier. As a result, electrons can easily traverse the interface, leading to a sharp increase in current and switching the device to LRS. Erasure is achieved by applying a negative voltage to drive the accumulated sulfide ions away from the interface [109].

### Performance Metrics for Artificial Synapses

**Fig. 7:**
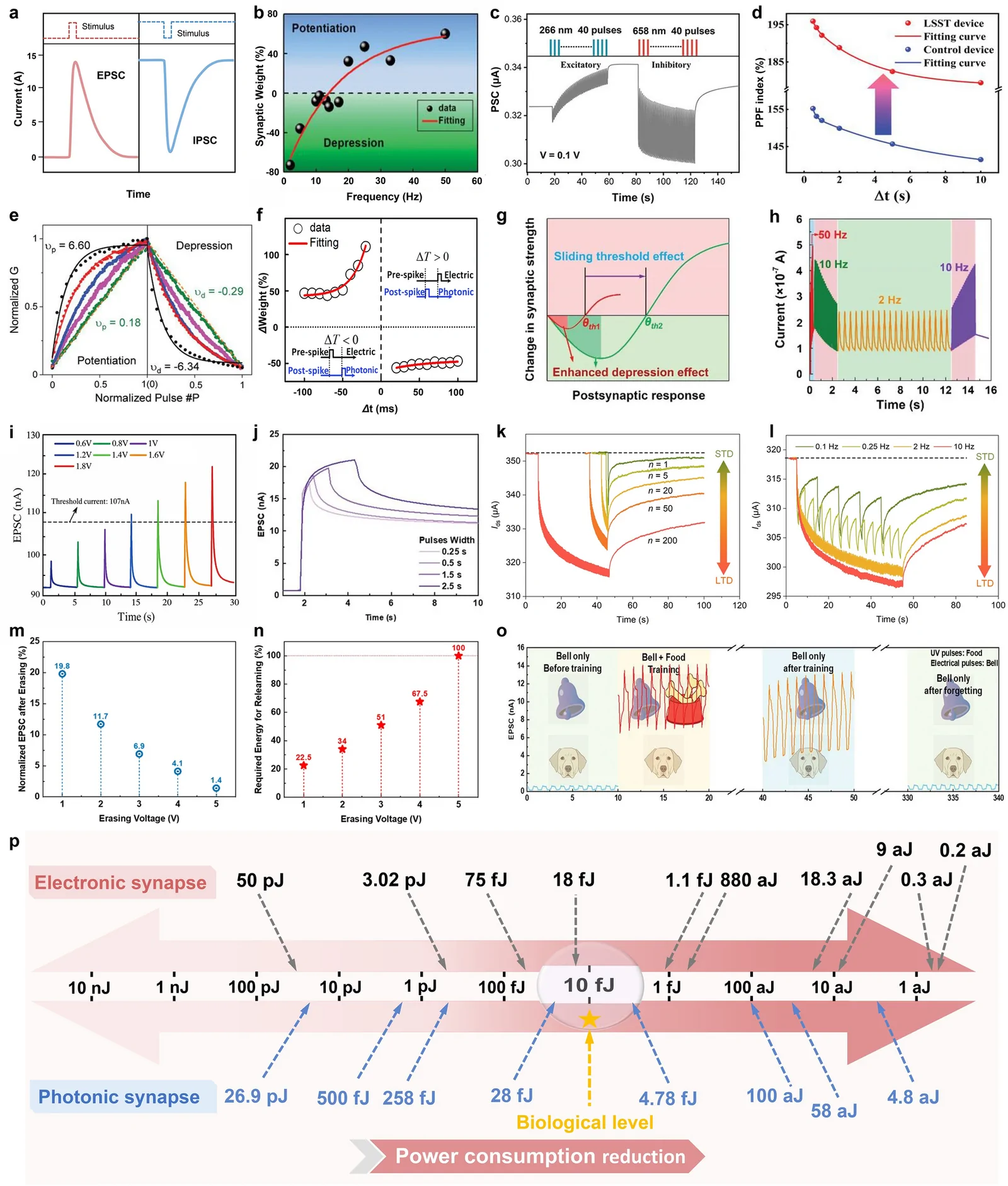
Performance metrics of artificial synapses. **a** Excitatory and inhibitory postsynaptic current (EPSC/IPSC) characteristics of artificial synaptic devices. **b** Synaptic weight as a function of presynaptic optical pulse frequency. Reproduced with permission [115]. Copyright (2019), Royal society of chemistry. **c** EPSC and IPSC responses under 266 and 658 nm optical stimulation. Reproduced with permission [116]. Copyright (2024), American Chemical Society. **d** High PPF behavior decomposition of a light-stimulated synaptic transistor. Reproduced with permission [117]. Copyright (2022), Wiley–VCH GmbH. **e** Normalized conductance for a potentiation and depression cycle under varying voltage pulse amplitudes. Reproduced with permission [12]. Copyright (2021), Wiley–VCH GmbH. **f** Synaptic weight changes as a function of spatiotemporally correlated inputs. Reproduced with permission [115]. Copyright (2019), Royal society of chemistry. **g** Biological BCM Curve Schematic. **h** A memristor response to consecutive spike trains with various frequencies. Reproduced with permission [124]. Copyright (2022), Wiley–VCH GmbH. **i**–**l** Synaptic plasticity implemented through modulation of various spike parameters, including pulse amplitude, duration, number, and rate. Reproduced with permission [132]. Copyright (2021), Wiley–VCH GmbH. Reproduced with permission [125]. Copyright (2021), Wiley–VCH GmbH. Reproduced with permission [133]. Copyright (2023), Wiley–VCH GmbH. **m** Normalized excitatory PSCs post-erasure at varying voltages. **n** Energy consumption for relearning after erasure at different voltages. **o** Classical conditioning simulation using ultraviolet light and electrical pulses as food and bell signals for associative learning. **p** Comparison of power consumption of various electronic and optoelectronic synapses in recent reports [12, 101, 121, 129, 130, 134–145]

In current artificial synaptic device research, performance evaluation criteria often rely on biological behavior fidelity. First, changes in the electrical properties of the postsynaptic membrane are a key indicator of synaptic transmission. Applying an action potential to the gate of a synaptic memtransistor (or to the electrodes of a memristor) or delivering optical stimulation to the channel layer can induce potential changes that are similar to biological postsynaptic currents (PSC). The modulation of PSC is categorized into excitation and inhibition (EPSC and IPSC), which can be readily replicated by bipolar-voltage-controlled [110] or unipolar-voltage amplitude-controlled [111] electronic synapses and optoelectronic hybrid synapses [112, 113] (refer to Fig. 7a). Notably, most photonic synapses designed for bionic vision robotics are unable to achieve simultaneous facilitation and inhibition through simple polarity switching. Researchers build their hopes on light-pulse frequency and energy to achieve dual photonic modulation capability [114–116]. The filtering effect of the MoS_2_ channel on different optical pulse frequencies was demonstrated by Jiang et al*.*, as shown in Fig. 7b [115]. Due to the differences in relaxation times of photogenerated carriers, high-frequency photons induce current superposition in MoS_2_ channels through the photoelectric effect, while the photogenerated carriers from low-frequency photons undergo prolonged trapping that manifests synaptic depression behavior. Figure 7c demonstrates a bidirectional modulation effect that implements synaptic excitation and inhibition, achieved by leveraging photon-energy-dependent control of carrier concentrations [116].

The emulation of synaptic plasticity constitutes another significant objective in the design of artificial synapses. It is the key to the hardware realization of brain-like learning and memory functions. Among them, the paired-pulse facilitation (PPF) and the paired-pulse depression (PPD) serve as fundamental behaviors of STP. High-precision synaptic devices capable of rapid visual processing and real-time information decoding rely on ultrahigh PPF indices. Han et al*.* reported that introducing an ultrathin carrier-modulation layer of hexagonal h-BN into graphene hybrid structures yields an ultrahigh PPF index (~ 196%) [117], as shown in Fig. 7d. The h-BN layer’s buffering effect on photogenerated carriers induces gradual Fermi-level lowering in graphene to enable this enhanced facilitation behavior. In addition, high linearity and symmetry in synaptic long-term potentiation (LTP) and long-term depression (LTD) responses are essential figures of merit for efficient backpropagation training of neural networks [118–120]. Simultaneously, this characteristic eliminates peripheral circuit-induced delays and power overhead, enabling ultralow-energy synaptic operation and low error rate in neural network implementations (Fig. 7e) [12]. The frequency, amplitude, and intersignal intervals of input stimuli often encode critical information. Multiple pulse parameter-dependent plasticity mechanisms, including spike-timing-dependent plasticity (STDP), spike-amplitude-dependent plasticity (SADP), spike-duration-dependent plasticity (SDDP), spike-number-dependent plasticity (SNDP), and spike-rate-dependent plasticity (SRDP), are necessary for emulation in artificial synaptic device development. STDP is a typical synaptic modulation method rooted in Hebbian learning rules [120–122]. The precise temporal sequence and interval between two pulses govern current intensity changes in the channel (or resistive switching layer), enabling fine-tuned control of interneuronal connection strength (as shown in Fig. 7f) [115]. However, a limitation of classical Hebbian learning rules lies in their lack of synaptic weight constraints, which may lead to an unexpected system crash. In contrast, the rule of Bienenstock–Cooper–Munro (BCM) introduces the global activity level of neurons [123]. As shown in Fig. 7g, h, identical inputs can elicit opposing effects under different historical frequencies, as the frequency threshold dynamically adapts based on prior activity levels. This adaptive regulation is a critical mechanism to prevent neural system damage caused by unbounded synaptic weight growth, which was experimentally confirmed in a MoS_2_/WSe_2_ heterostructure memtransistor [124]. Synaptic weight modulation is further contingent upon the amplitude and duration of the applied pulse. The amplitude determines the write energy intensity, while the pulse duration affects the kinetic processes, including carrier transport, phase transition, or polarization reversal, among others (Fig. 7i, j). In studies of SNDP and SRDP, repeated high-frequency stimuli drive synapses through iterative learning–forgetting–relearning cycles, ultimately achieving long-term memory (LTM) consolidation (Fig. 7k, l). Based on the aforementioned plasticity mechanisms, certain complex biological neural activities, such as immune responses and conditioned reflexes, can be emulated at the hardware level [125]. As shown in Fig. 7m, n, after 400 negative pulses of learning, different amplitudes of positive electrical signals were applied for erasure. Higher amplitudes resulted in lower residual PSC values post-erasure, consequently demanding greater energy expenditure during the relearning phase. This relearning process is similar to a form of sensitization, a protective mechanism by which organisms respond to injury. Another prominent example of emulation is the Pavlov’s dog conditioned reflex experiment, in which electrical and optical stimuli represent the neutral cue (bell) and the unconditioned signal (food). The coordination of these pulses by the artificial synapse enables it to learn to respond to the neutral cue with a change in conductance, demonstrating associative learning through training, acquisition, and forgetting processes (Fig. 7o).

Notably, reducing energy consumption is crucial to driving the development of brain-inspired computing technologies. This requires a fundamental reduction in the operating voltage and response current, alongside an enhancement in the response speed of devices. To date, artificial synaptic devices constructed from 2D materials have achieved energy consumption at the femtojoule (fJ) or even lower, which is comparable to that of a single biological synaptic event (~ 10 fJ) [126]. In conductive filament synapses, the low-energy barrier channels provided by the interlayer gaps of 2D materials enable ions to move by overcoming weak van der Waals forces, forming conductive filaments as fine as atomic chains, which can reduce the energy consumption of a single operation to the zeptojoule (1 zJ = 10^–21^ J) level [32]. 2D ferroelectric and spintronic synaptic devices offer a switching route by leveraging the ultralow-energy motion of domain walls at the atomic scale. They operate at reduced voltages, avoiding the high coercive fields of conventional ferroelectric films and greatly diminishing the power demand in synaptic weight modulation. The low-dimensional architecture of phase-change materials enables attojoule-level switching energy by drastically reducing the thermal energy input and current density required to induce the phase transition. In addition, heterostructure engineering and device architecture design based on 2D materials can also effectively reduce the power consumption of synaptic devices. For instance, in electrolyte-gated synaptic devices, the ultrathin 2D channel offers exceptional gate control efficiency. Meanwhile, the electric double layer formed at the electrolyte interface features a high capacitance per unit area. Thus, such transistors can induce a high carrier density in the channel under low gate voltages, enabling low-voltage and high-precision conductance modulation. [106]. The floating-gate structure has also been widely demonstrated to exhibit low operating voltage and superior charge storage capability [99, 127, 128]. A novel WSe_2_/MoS_2_ heterojunction channel achieves a steeper subthreshold swing compared to monolayer channels. When the channel simulates synaptic behavior under 500 ns electrical stimulation pulses, its energy consumption per potentiation event reaches as low as 9 aJ (1 aJ = 10^–18^ J) [129]. However, the non-ideal operation speed of floating-gate transistors poses a fundamental limitation to minimizing energy consumption per operation. The Han’s team ingeniously proposed a polarized tunneling transistor structure based on an MoS_2_/Trap/PZT heterojunction. Without a tunneling layer, it achieves an ultrafast operation speed of 20 ns, enabling a synaptic weight update energy consumption of only 0.2 aJ [130]. Additionally, tunnel field-effect transistors utilize a gate-controlled PN junction to enable band-to-band tunneling, which allows for an ultra-steep subthreshold swing and a significant reduction in operating voltage [131].

The relationship between power consumption and device size cannot be overlooked either. For example, in interface-dependent memristors, miniaturizing the active region can effectively enhance the local electric field intensity and provide more efficient driving force. For devices that the conductance modulation within the functional material, the benefits of miniaturization mainly come from shortening the physical path for ion migration or charge transport, thereby reducing the operating voltage and response time. Moreover, high-density integration reduces the average interconnect distance between units, lowering the parasitic capacitance and resistance of the interconnects, resulting in reduced dynamic energy consumption for signal transmission. However, the continuous miniaturization still faces problems such as excessive leakage current, increased contact resistance, thermal management, and amplification of process fluctuations. Therefore, a rational balance should be made among device size, performance, and reliability. A systematic comparison of synaptic energy consumption across representative studies is presented in Fig. 7p. The star symbol marks the biological synaptic benchmark (10 fJ per synaptic event), highlighting the breakthrough potential of 2D materials artificial synaptic designs in energy efficiency. Table 2 presents a summary of the key mechanisms and performances of 2D material-based synaptic devices.

**Table 2:** Mechanisms and performances of artificial synapse devices based on 2D materials

Mechanism	Materials	Construction	Synaptic plasticity	On/off ratio	Energy or power consumption (per spike)	States	Symmetric ratio/linearity	Availability of stimuli	Application	References
Conductive Filament	Au/h-BN/GaN	2-Terminal	LTP^*a*^/LTD/Multi-state modulation	~ 6.5	/	6	nonlinearity factors = 0.487/2.012	Electric	MNIST image recognition	[146]
	Cu/HfO_x_/BP/Pt	2-Terminal	STP/LTD/PPF/SNDP	~ 10^2^	/	4	/	Electric + Optical	Image recognition/Artificial Vision Systems	[147]
	Ag/Bi_2_O_2_Se/Au	2-Terminal	LTP/PPF/STDP	~ 10^3^	3.02 pJ	/	asymmetric ratio = 0.29	Electric	MNIST image recognition	[121]
	Ag/MoTe_2_/ITO	2-Terminal	STDP/PPF/LTP/LTD/SNDP	8	74.2 pJ	/	/	Electric	Decimal arithmetic function	[148]
	Cu/2H-MoTe_2_/Si	2-Terminal	PPF/LTP/LTD/STDP	~ 13	0.86 μW	/	linearity = 0.93	Electric	Decimal counting/adding functions	[149]
	Ag/GeSe/Au	2-Terminal	LTP/LTD/PPF	~ 700	3.5 nJ/562 pJ (excitation/inhibition)	180	/	Electric	Handwritten digit recognition	[150]
	Pd/WS_2_/Pt	2-Terminal	PPF/STDP/SRDP/SDDP/STDP/PPF	/	299.8 fJ/125.6 fJ (excitation/inhibition)	≥ 4	/	Electric	/	[151]
	Au/TiO_x_/MoS_2-x_O_x_/Au	2-Terminal	LTP/LTD/Multi-state modulation	~ 7	/	64	*C*_LTP_/*C*_LTD_^*b*^ = 1.7%/1.3%	Electric	MNIST image recognition	[152]
Phase transition	Au/Li_x_MoS_2_/Au	2-Terminal	Memristive switching	~ 50	/	/	/	Electric	/	[80]
	Ag–Ni/MoTe_2_/Ag–Ni	2-Terminal	Memristive switching	10^8^	150 aJ	/	/	Electric + Strain	/	[153]
	Au/Ag-intercalated MoTe_2_/Au	2-Terminal	LTP/LTD/Multi-state modulation	2 × 10^5^	/	~ 80 states per μm^2^	nonlinearity factors = 0.5 ~ 0.6	Electric	MNIST image recognition	[154]
	Au/Cu_2_S/Au	2-Terminal	LTP/LTD/Multi-state modulation	10^2^ ~ 10^4^	2.64 pJ	≥ 5	/	Electric	Gesture recognition	[155]
Ferroelectric effect	Au/SnS_2_/CuInP_2_S_6_/h-BN/Au Au (BG^*c*^)	3-Terminal	PPF/SDDP/SNDP/LTP/LTD	> 10^6^	3.06 pJ	/	/	Electric	Intelligent Vehicle Target Recognition / Robotic Manipulation	[156]
	Graphene/CuVP_2_S_6_/Graphene	2-Terminal	LTP/LTD/PPF/PPD/SADP/SNDP/SDDP	/	/	214	/	Electric + Optical	Hand written letter recognition/Neural machine translation	[157]
	Au/SnSe/NSTO	2-Terminal	PPF/PPD/SNDP/SADP/STDP	/	66 fJ	/	/	Electric	/	[94]
	Au/α-In_2_Se_3_/Au Au (TG^*d*^) P^++^-Si (BG)	3-Terminal	LTD/LTP/SADP/SRDP	> 10^3^	234 fJ/40 fJ (excitation/inhibition)	/	/	Electric + Thermal	Iris recognition and classification	[96]
	Au/NbOI_2_/Au	2-Terminal	PPF/SADP/SNDP/SRDP/SDDP	/	/	/	/	Optical + Strain	Fingerprint Image Enhancement and Recognition	[158]
Spintronics	PtCx/Fe_3_GeTe_2_/PtCx	2-Terminal	LTP/Multi-state modulation	/	/	8	/	Electric	MNIST image recognition	[97]
	Au/Bi_2_Te_3_/CrTe_2_/Au	2-Terminal	LTP/LTD/Multi-state modulation	10	10 ~ 100 fJ	18	linearity error = 4.19%	Electric	MNIST image recognition	[98]
Charge modulation	Au/MoS_2_/GaPS_4_/Au n-Si (BG)	3-Terminal	LTP/LTD/PPF/SADP/SDDP/SNDP	10^5^	/	8	/	Electric + Optical	MNIST image recognition	[159]
	Au/graphene/Au PVDF-TrFE (n-Gel Gate Dielectrics) Au (BG)	3-Terminal	LTP/LTD/PPF/SADP/SRDP/SNDP/SDDP	/	/	/	/	Strain	Electronic skin	[160]
	Au/InSe/Au	2-Terminal	PPF/SADP/SRDP/STDP/BCM	/	1.1 fJ	/	/	Electric	Classical conditioning/Image edge recognition	[135]
	Au/MoS_2_/Au p-Si (BG)	3-Terminal	PPF/SADP/SNDP/SDDP	> 10^5^	~ 63 pJ/ ~ 1.85 nJ	/	/	Electric + Optical	Classical conditioning/signal self-denoising	[161]
	Au/BP/Au ITO (TG) HfO_2_ (FG^*e*^)	3-Terminal	Multi-state modulation	~ 200	/	≥ 8/36 (Electric/optical)	/	Electric + Optical	Imaging with in-sensor computing for edge detection/image recognition	[162]
	Au/MoS_2_/Au Graphene (FG)	3-Terminal	LTP/LTD/SVDP/SDDP/SNDP	10^8^	18 fJ	131	nonlinearity factors = 0.18/-0.29	Electric	MNIST image recognition	[12]

^*a*^*LTP* Long-term potentiation, ^*b*^*C*_*LTP*_ and *C*_*LTD*_ Cycle-to-cycle variations of LTP and LTD process, ^*c*^*BG* Bottom gate, ^*d*^*TG* Top gate, ^*e*^*FG* Float gate

## 2D Materials-based Reconfigurable Artificial Neurons and Synapses

**Fig. 8:**
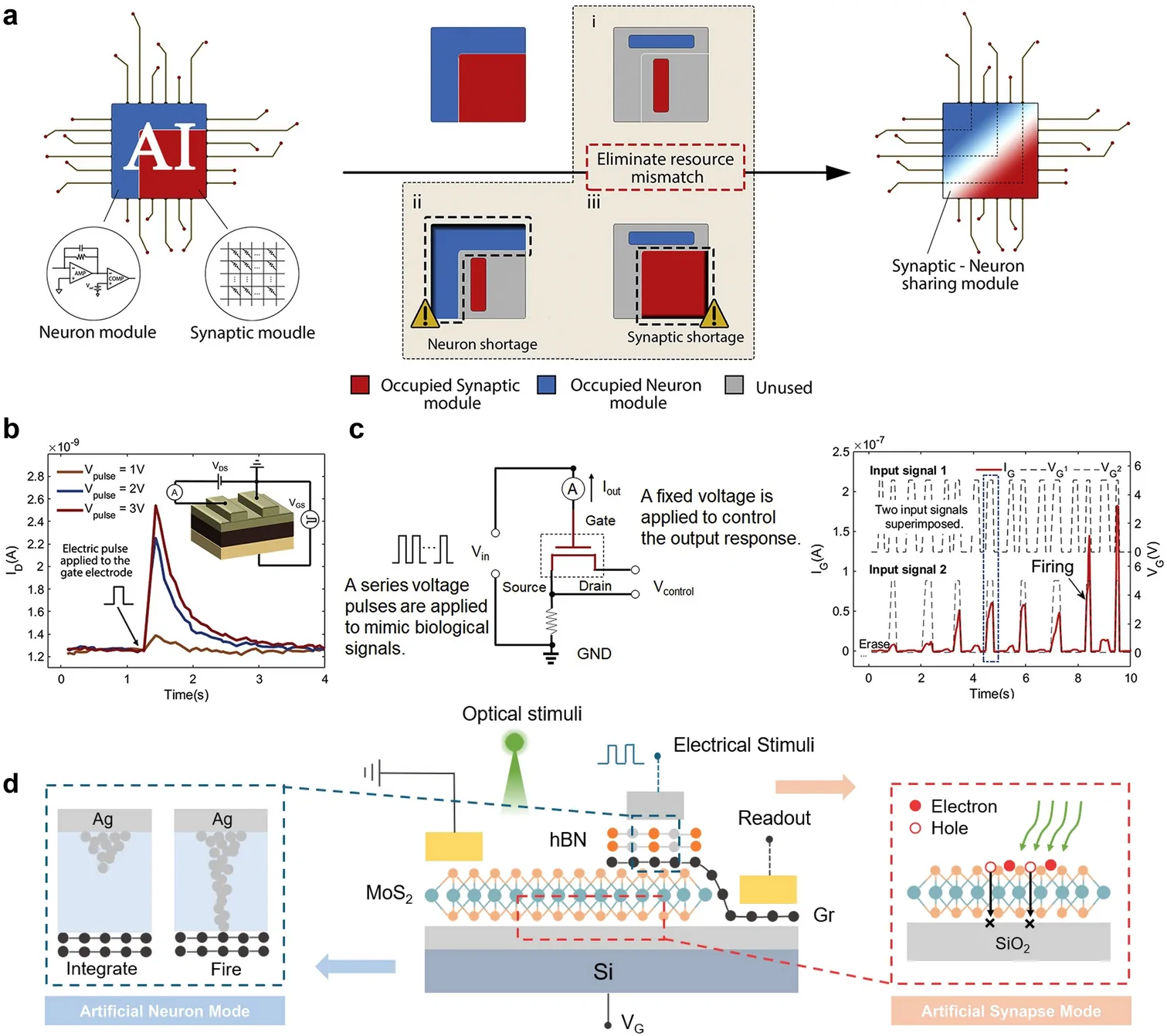
**a** Three kinds of hardware mismatch phenomena when performing different tasks. Therefore, it is imperative to eliminate the boundary between the two modules, establishing a neuromorphic chip with a neuron–synapse shared module. **b** EPSC triggered by electric pulses applied to the gate. **c** Circuit diagram of a reconfigurable device with neuronal functionality and spike pulses after integration of input information in the neuron model. Reproduced with permission [163]. Copyright (2022), Elsevier. **d** Structure of the reconfigurable neuromorphic unit with multimodal neuron and synapse key functions [164]

The fundamental building blocks of classical brain-inspired systems are interconnected neuron and synapse modules. Dedicated neuromorphic devices demonstrate notable advantages in the pursuit of extreme performance and low latency. By contrast, RNDs are specifically suitable for scenarios where hardware resources and algorithmic requirements are not fully aligned, enabling adaptive functional transformation and module sharing, as illustrated in Fig. 8a [163]. In the design of RNDs, the intuitive difference between neuron and synapses modes lies in their electrical dynamic characteristics. Abrupt and volatile properties can induce the generation of pulse signals, while gradual and non-volatile properties can reflect the superposition effect of signals. The reconfigurability of these dynamic properties relies on the orchestration of structural design, external control, and internal mechanism. The abundant and tunable physical properties of 2D materials provide enhanced flexibility in the implementation pathways for RNDs. Accordingly, we categorize the implementation pathways into three types based on their dominant factors: terminal programming, input parameter regulation, and materials property modulation.

### Terminal Programming

The terminal programming strategy enables a single device to invoke different modules or units by distinct electrodes. These modules or units are partially overlapping in structure but become complete small units once invoked, allowing them to execute neuronal spike firing and synaptic weight updating separately. Therefore, dynamic allocation of brain-inspired computing is realized at the hardware level. Such programmatically wiring strategy is implemented in the MXene-based integrated multi-neuromorphic-functional synaptic transistor (SNST) proposed by Zhang et al*.* [163] When pulses are applied to the gate, the electrical response of the channel is realized through proton migration in the polyvinyl alcohol electric double layer. This behavior simulates the excitatory postsynaptic current, representing changes in synaptic weight (Fig. 8b). In its neuron mode, the SNST is similar to a two-terminal memristor (formed by the gate and source). By applying different gate voltages, Ag⁺ ions can be adsorbed by the gate dielectric layer (doped MXene) surface to promote the formation of conductive filaments. Once the gate voltage is removed, the Ag filaments naturally dissolve and break, enabling the critical TS characteristic of neurons (Fig. 8c). Furthermore, the architecture holds the potential to achieve functional diversity by incorporating mixed-mode inputs and additional terminals. In Fig. 8d, light illumination modulates the conductance of the MoS_2_ channel to exhibit the plasticity of the optoelectronic synapse. Induced by top-gate pulses, the device functionally resembles a memristor with a spiking encoding mechanism in the vertical direction. Meanwhile, the electrical inputs from the bottom gate and drain, along with optical pulses, enable the nonlinear integration of dendrites [164]. Certainly, this reconfigurable technology has certain limitations: large-scale multiple-gating structures lead to increased system complexity and manufacturing costs, as well as potential signal crosstalk and increased parasitic capacitance.

### Input Parameter Regulation

**Fig. 9:**
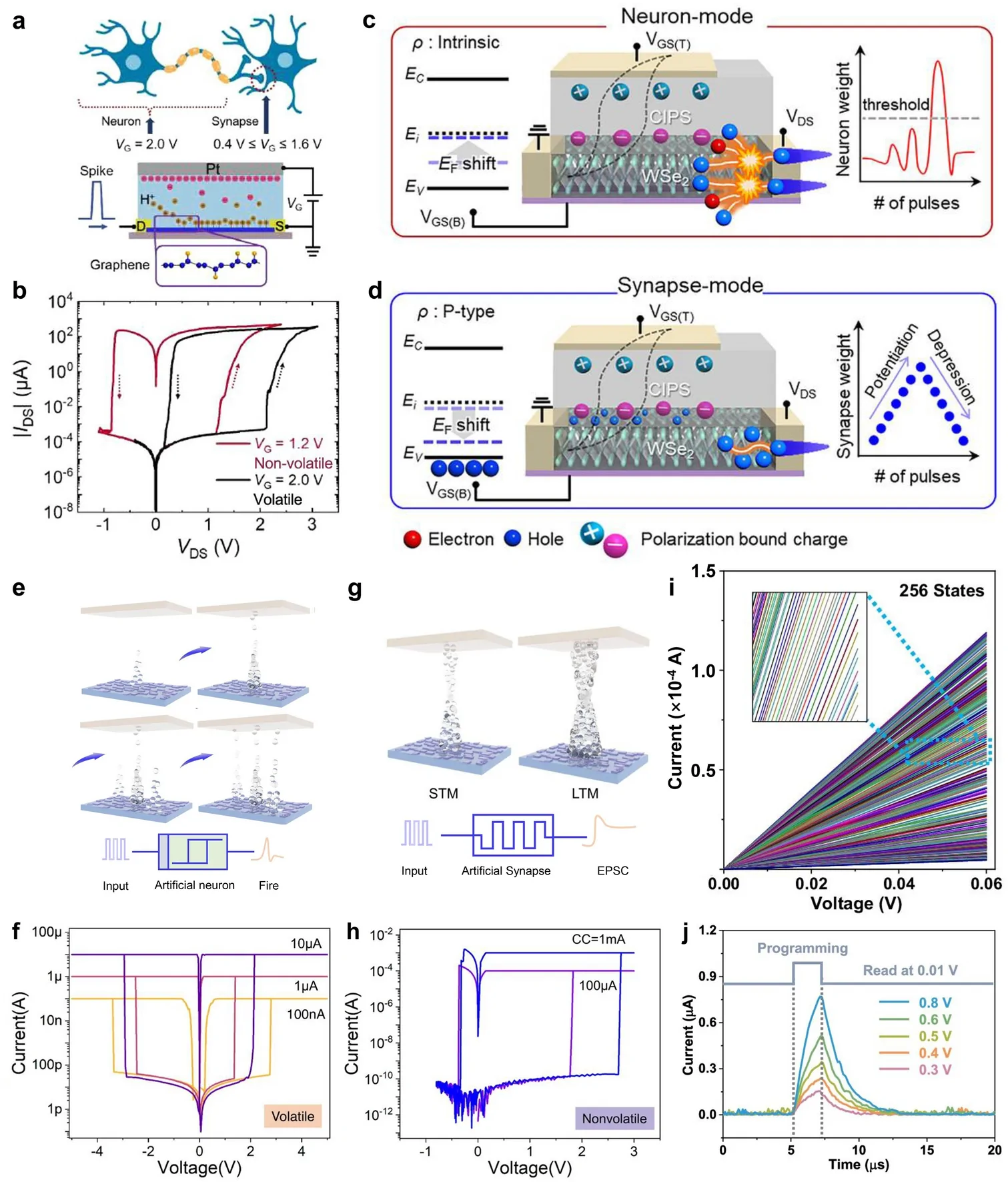
**a** Schematic diagram of hydrogenation reactions between graphene lattices and hydrogen ions and a corresponding biological neuron model. **b** Non-volatile and volatile gate-controlled memristive behaviors in the electrochemical graphene transistors. Reproduced with permission [165]. Copyright (2024), American Chemical Society. **c-d** Mechanism diagram of neuron mode and synapse mode in CuInP_2_S_6_/h-BN/WSe_2_ heterostructure transistor. Reproduced with permission [17]. Copyright (2025), Wiley–VCH GmbH. **e** Mechanisms of LIF neuron behaviors in Ag/MoS_2_/HfAlO_x_/CNT textile memristor under consecutive pulse stimulation. **f** Volatile resistive switching behaviors under low current compliance. **g** Short- and long-term memory characteristics in artificial synapses correspond to weak and strong conductive filaments, respectively. **h** Non-volatile resistive switching behaviors under high current compliance. Reproduced with permission [76]. Copyright (2022), Spring Nature. **i** Tunable multi-level conductance states. **j** Evolution of volatile current in a ZnPS_3_ memristor under single electrical pulse stimuli of varying amplitudes. Reproduced with permission [16]. Copyright (2025), Spring Nature

The input parameter regulation reconfigurable strategy achieves functional differentiation of neuromorphic devices by modulating material properties or carrier transport behaviors through variations in external control signals (e.g., voltage amplitude, compliance current, pulse width, and others). For example, based on the reversible electrochemical reaction between graphene and hydrogen ions, Yu et al*.* ingeniously utilized gate voltage amplitude to precisely isolate volatile and non-volatile states, as shown in Fig. 9a, b [165]. In graphene transistors, both gate voltage (*V*_gs_) and source–drain voltage (*V*_ds_) are critical factors regulating channel conductivity. For artificial synapse emulation, when the *V*_gs_ is fixed lower than the hydrogenation voltage (*V*_H_, ~ 1.8 V) and the graphene channel is in HRS, the *V*_ds_ determines the effective gate voltage (*V*_g,Eff_) applied to graphene, indirectly influencing the hydrogenation reaction process. *V*_g,Eff_ decreases with increasing *V*_ds_. Once it falls below the dehydrogenation voltage (*V*_DH_, ~ 0 V), the corresponding graphene segment undergoes dehydrogenation and sets up a channel to LRS. Conversely, if *V*_g,Eff_ remains sufficiently high, the graphene segment becomes hydrogenated, resetting the channel to HRS. Artificial neuron functionality is achieved by setting *V*_gs_ higher than *V*_H_, where the device exhibits volatile alternation between HRS and LRS through *V*_ds_ modulation. Upon retracting *V*_ds_ to 0 V, the hydrogenation reaction spontaneously (*V*_gs_ > *V*_H_) restores the HRS state to implement LIF neuron functionality. A similar electrostatic modulation scheme has also been implemented in a reconfigurable CuInP_2_S_6_/h-BN/WSe_2_ heterostructure-based transistor, which selectively activates the dominant physical mechanism through back-gate electrostatic modulation of channel carrier density [17]. In neuronal mode, the Fermi level (*E*_F_) of WSe_2_ is tuned to its intrinsic level by a global back-gate voltage (*V*_BG_). When repeated stimulus signals are applied to the top gate, the ungated region bears the full magnitude of the source–drain voltage. Within this region, carrier acceleration induces avalanche multiplication, leading to a rapid current spike (Fig. 9c). Switching to synaptic mode, the *E*_F_ is modulated closer to the valence band by a more negative *V*_BG_. The high carrier density results in enhanced carrier interactions and electrostatic screening, which suppresses impact ionization. At this stage, non-volatile memory is dominated by ferroelectric polarization (Fig. 9d).

In addition, the mode reconfiguration between volatile neurons and non-volatile synapses can also be achieved by using programmed external electrical stimuli to control the growth and rupture dynamics of the filaments. The typical *I-V* characteristics reveal that a low compliance current leads to the formation of a few fragile conductive filaments within the functional layer. These filaments spontaneously rupture after the power removal, thereby exhibiting volatile TS that corresponds to neurons’ firing and rapid resetting following threshold excitation. In contrast, robust filaments tend to form at a higher compliance current. The filament remains stable after the voltage is withdrawn, thus enabling a non-volatile transition in the resistive state of the device, which corresponds to the long-term memory effect in synapses. As illustrated in Fig. 9e–h, an Ag/MoS_2_/HfAlO_x_/carbon nanotube (CNT) textile memristor network was developed by Chen’s group to verify this reconfiguration method [76]. Under low-amplitude, narrow-width pulse stimulation, weak conductive filaments are formed to modulate the switching behavior of the functional layer. A single reconfigurable memristor suffices to implement neuronal integrate-and-fire functionality at an energy cost of only 1.9 fJ per event. In synaptic mode, high-amplitude wide pulses promote non-volatile filament formation, where short-term or long-term memory effects correlate with the strength of conductive filaments in the memristor. Similarly, through the control of metal conductive filaments, 256 different non-volatile conductive states and volatile switches with energy consumption as low as 143 aJ/peak were achieved in memristors with layered single-crystal ZnPS_3_ as the functional layer (Fig. 9i, j) [16]. Beyond the electrically dominated dual-mode reconfiguration of neurons and synapses, Yan’s group has recently ingeniously introduced optical pulses as a parallel control scheme [57]. Under this framework, reversible switching between volatile and non-volatile states is achieved by modulating optical parameters, specifically the power density and pulse width of the light input. This approach holds the potential to mitigate undesirable crosstalk and facilitate the construction of all-optical modulation neural networks.

The advantage of the aforementioned reconfigurable strategy lies in its ability to achieve functional switching solely through signal modulation, eliminating the need for complex multi-terminal designs and significantly reducing system complexity. However, it should be noted that applications may be limited by signal crosstalk and material degradation under high-frequency switching conditions.

### Materials Property Modulation

**Fig. 10:**
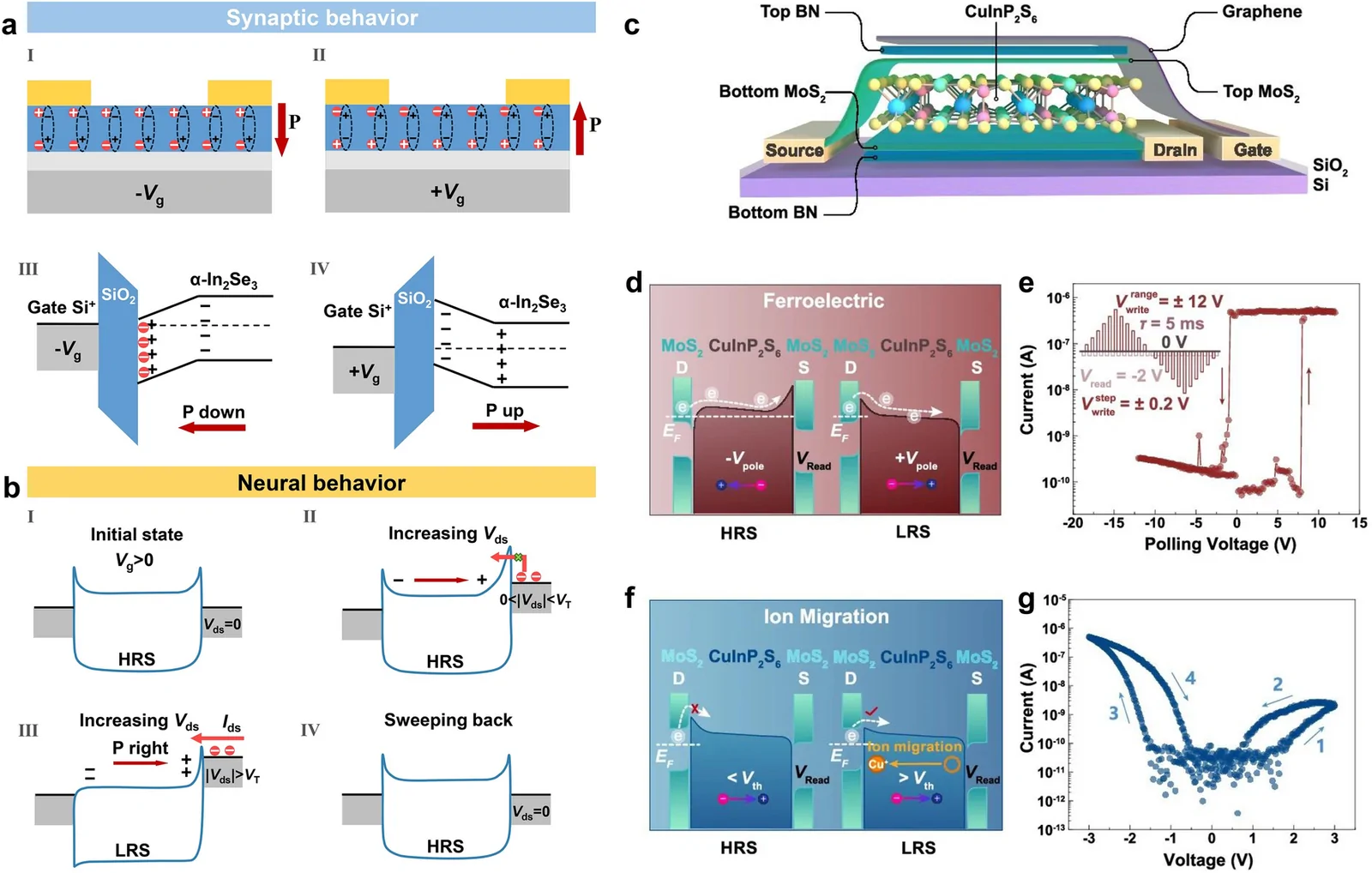
**a** α-In_2_Se_3_ ferroelectric field-effect transistor and corresponding band diagrams under positive or negative gate voltages for synaptic weight modulation. **b** Energy band diagram illustrating the operational mechanism of reconfigurable neuromorphic devices functioning as neurons. Reproduced with permission [10]. Copyright (2023), AIP Publishing. **c** MoS_2_/CuInP_2_S_6_/MoS_2_ refreshable memristor device structure. **d** Energy band diagram of the device in HRS and LRS states under non-volatile ferroelectric polarization mode. **e** A typical ferroelectric memory window characteristic curve. **f** Energy band diagram of the device in HRS and LRS states under volatile ion migration mode. **g** Volatile *I-V* characteristics of the device under a fixed gate voltage of − 4 V. Reproduced with permission [166]. Copyright 2025, Springer Nature

The third strategy ingeniously leverages the intrinsic properties of materials for reconfigurable operations. Compared to bulk materials, 2D materials exhibit a broader spectrum of tunable properties. The physical origin of this exceptional tunability lies in the direct manipulability of its electronic structure, ionic distribution, and even crystal phase, leading to significant changes in its physical properties. These adjustable intrinsic characteristics establish the physical foundation for high-performance reconfigurable devices. A typical example is the utilization of the polar nature of α-In_2_Se_3_ to achieve switching between synaptic and neuronal modes. The update of synaptic weight was controlled via vertical OOP polarization modulation according to the amplitude, width, and number of the gate pulse, which further affects the movable charges (Fig. 10a) [10]. The *V*_ds_-tuned parallel IP polarization and Schottky barrier modulate the carriers of a lateral memristor structure, enabling integrated-firing characteristics, as illustrated in Fig. 10b. Moreover, Chen et al*.* proposed a refreshable memristor based on CuInP_2_S_6_, achieving neural reuse through dynamic allocation of ferro-ion phases (Fig. 10c) [166]. The ferroelectric polarization in CuInP_2_S_6_ stems from the off-center ordering of Cu⁺ ions. A series of source–drain pulses triggers polarization switching, and following voltage withdrawal, the electric dipoles remain stabilized in a new orientation, thereby achieving non-volatile memory effects (Fig. 10d, e). The volatile mechanism originates from the accumulation of Cu⁺ ions at the interface following long-range migration, which modulates the energy barrier. Upon removal of the electric field, these ions rapidly relax back to their original positions through spontaneous recovery (Fig. 10f, g).

Furthermore, various material-related factors, including electrode chemical activity, defect, and interface properties, etc., may also critically influence the overall performance of neuromorphic devices: (i) The chemical activity and ionic migration capability of the electrode material collectively regulate its dynamic evolution behavior. As a result, the filament growth and stability are fundamentally altered, thereby dictating distinct switching behaviors [167]. Moreover, the filament’s conductivity (determined by the filament material and density) governs Joule heating dissipation, which directly correlates with spontaneous conductive filament rupture [168]. (ii) Doping basic dielectric materials with specific elements can modify their properties to achieve functional differentiation. For instance, doping in phase-change materials alters the electronic structure, thereby suppressing the rapid metal–insulator transition (MIT) and converting it into a non-volatile process [169]. Alternatively, doping induces changes in the vacancy formation energy, ion migration barriers, and local electric field distribution within the dielectric material, leading to modifications in the stability of conductive filaments. (iii) The internal physical processes of a device can be dominated by interface engineering and barrier modulation between films. For instance, incorporating an ultrathin barrier layer can modulate the efficiency of ion/charge injection, thereby determining the stability of the conductive filaments [170]. Volatile or non-volatile switching behavior can also be determined by modulating the Schottky barrier height, the concentration, and mobility of defects [171]. These approaches would facilitate further exploration of novel neuromorphic reconfigurable strategies. Herein, we summarize the characteristics of reconfigurable artificial neurons and synapses based on 2D materials, as systematically presented in Table 3.

**Table 3:** Mechanisms and characteristics of reconfigurable neuron and synapse devices based on 2D materials

Materials	Type	Construction	Reconfigurable mechanism	Neuron property			Synapse property			Energy or power consumption (per spike)	References
				Model	Threshold (V)	On–off ratio	Plasticity	On–off ratio	States		
ITO/(PVA-Mxene)/ITO Ag (BG)	Terminal programming	3-Terminal	EDL effect/ECM	LIF	8	/	SADP/LTP^*d*^/LTD^*e*^	~ 24	25	/	[163]
Au/h-BN/Graphene/MoS_2_/Au Ag (TG) Si (BG)		3-Terminal	photovoltaic effect/ECM/photogating effect	IF	0.18	~ 10^4^	PPF/SRDP	/	/	37.5 nW for neuron	[164]
EGaIn/GaO_x_/MXene or rGO/W n^+^-Si (BG)		3-Terminal	charge transport/ECM/	IF	3.56	/	LTP/LTD	/	4	/	[174]
Ag/MoS_2_/HfAlO_x_/CNT	Input parameter regulation	2-Terminal (electronic textiles)	ECM (adjusting the current compliance)	LIF	~ 2	10^6^	PPF/SVDP/LTP/LTD	10^8^	6	1.9 fJ for neuron	[76]
Ag/MoS_2_/Au (paper-based)		2-Terminal	ECM (adjusting the current compliance)	LIF	~ 0.4	10^5^	LTP	10^7^	/	50 pW for neuron	[175]
Ag/ZnPS_3_/Au		2-Terminal	ECM (adjusting the current compliance)	LIF	~ 0.2	10^6^	SNDP/LTP/LTD	~ 5 × 10^8^	256	143 aJ for neuron/ 28 fJ for synapse	[16]
Au/γ-Cu_2_S/Au		2-Terminal	γ and β phases transition in Cu_2_S/Cu_1.8_S conductive path (adjusting the current compliance)	/	< 0.6	~ 10	/	~ 10^2^	/	/	[176]
Ag/Bi_2_SeO_5_/Au		2-Terminal	ECM (adjusting the current compliance)	/	0.3	10^7^	LTP/LTD/SNDP	10^8^	11	1.16 pJ for synapse	[177]
Ag/SiO_2_/FA_2_PbI_4_/Pt nano particles/ITO		2-Terminal	ECM (adjusting the current compliance)	LIF	< 2	~ 10^4^	PPF/STDP/LTP/LTD	10^6^	/	~ 10 fJ for neuron/ ~ 20 nJ for synapse	[178]
Au/Graphene/Au HIE ^*a*^ (Dielectric) Pt (TG)		3-Terminal	gate-controlled electrochemical reactions	LIF	< 2.2	10^6^	LTP/LTD	/	≥ 5	/	[165]
Au/WSe_2_/Au CuInP_2_S_6_ (Dielectric) Au (TG) p^+^-Si (BG)		3-Terminal	impact ionization and ferroelectric polarization	LIF	1.13	> 10^6^	LTP/LTD	~ 6	/	/	[17]
Pd/ZnO/Graphene/SiO_2_/Si		2-Terminal	pulse stimulation mode	/	~ 2	~ 10^2^	LTP	~ 10^2^	/	/	[57]
ITO/Al_2_O_3_/HfSe_2_ /Al_2_O_3_/p-Si	Materials property modulation	2-Terminal	charge trapping/*V*_FB_^*b*^ shift and memcapacitor	LIF	/	/	LTP/LTD	/	8	/	[179]
Au/α-In_2_Se_3_/Au p-Si (BG)		3-Terminal	polarization effects	LIF	− 4.4	10^3^	STDP/SADP/LTP/LTD	10^3^	/	/	[10]
Au/MoS_2_/CuInP_2_S_6_/MoS_2_/Au Graphene (TG)		3-Terminal	ferroelectric polarization /ion migration	/	− 1.5	< 10^2^	SNDP/LTP/LTD	~ 10^4^	16	/	[166]
Ni/WSe_2_/Ni Hf_0.5_Zr_0.5_O_2_ (Dielectric) W (FG/BG)		3-Terminal	ferroelectric polarization switch/ electron self-compensation	LIF	/	/	SNDP/PPF/LTP/LTD	> 10^7^	/	/	[180]
Ni/MoS_2_/Ni Hf_0.17_Zr_0.83_O_2_ (Dielectric) W (FG/BG)		3-Terminal	AFE ^*c*^ switching/charge trapping	LIF	/	/	LTP/LTD/SADP/SDDP	10^7^	/	0.1 pJ for neuron/ 0.15 p J for synapse	[181]

^*a*^*HIE* Hydrogen ion electrolyte, ^*b*^*V*_FB_: Flat-band voltage, ^*c*^Antiferroelectric, ^*d*^*LTP* Long-term potentiation, ^*e*^*LTD* Long-term depression

It is also worth noting that reconfigurable devices extend beyond neurosynaptic dynamics to broader functionalities. In a study by Peng et al., a single-gate MoTe_2_ device was programmed via a gate-voltage-controlled gradient doping strategy to operate as a polarity-switchable diode, a memory element, an in-memory Boolean logic gate, and an artificial synapse [172]. Further advancing cross-modal functionality, Chen et al. demonstrated reconfigurable capability between optical switch–synapse and optical switch–storage modes in an optoelectronic device. This was achieved through an electrode-inserted structure, the synergistic properties of Graphene/VO_2_ heterostructures, and an external bias control [173]. In summary, achieving reconfigurable device functionality necessitates in-depth exploration and co-optimization of precise structural design, dynamic input parameter regulation, and the full exploitation of intrinsic material properties, thereby promoting the development of multi-task adaptive integrated circuits and systems.

## System-Level Implementations of Artificial Neurons and Synapses

The continuous emergence of novel dedicated and reconfigurable artificial synapses and neurons based on 2D materials has established a rich material foundation and diverse device prototypes for integrated neuromorphic development. Based on individual devices, interconnection of artificial neurons and synapses, or with biomimetic sensors and actuators, forms end-to-end sensory neuromorphic systems. The system integrates dynamic environmental perception from multiple sensory modalities, enabling real-time learning and adaptation to achieve complete perception-cognition process.

### Neuron–Synapse Integration and Interconnection

**Fig. 11:**
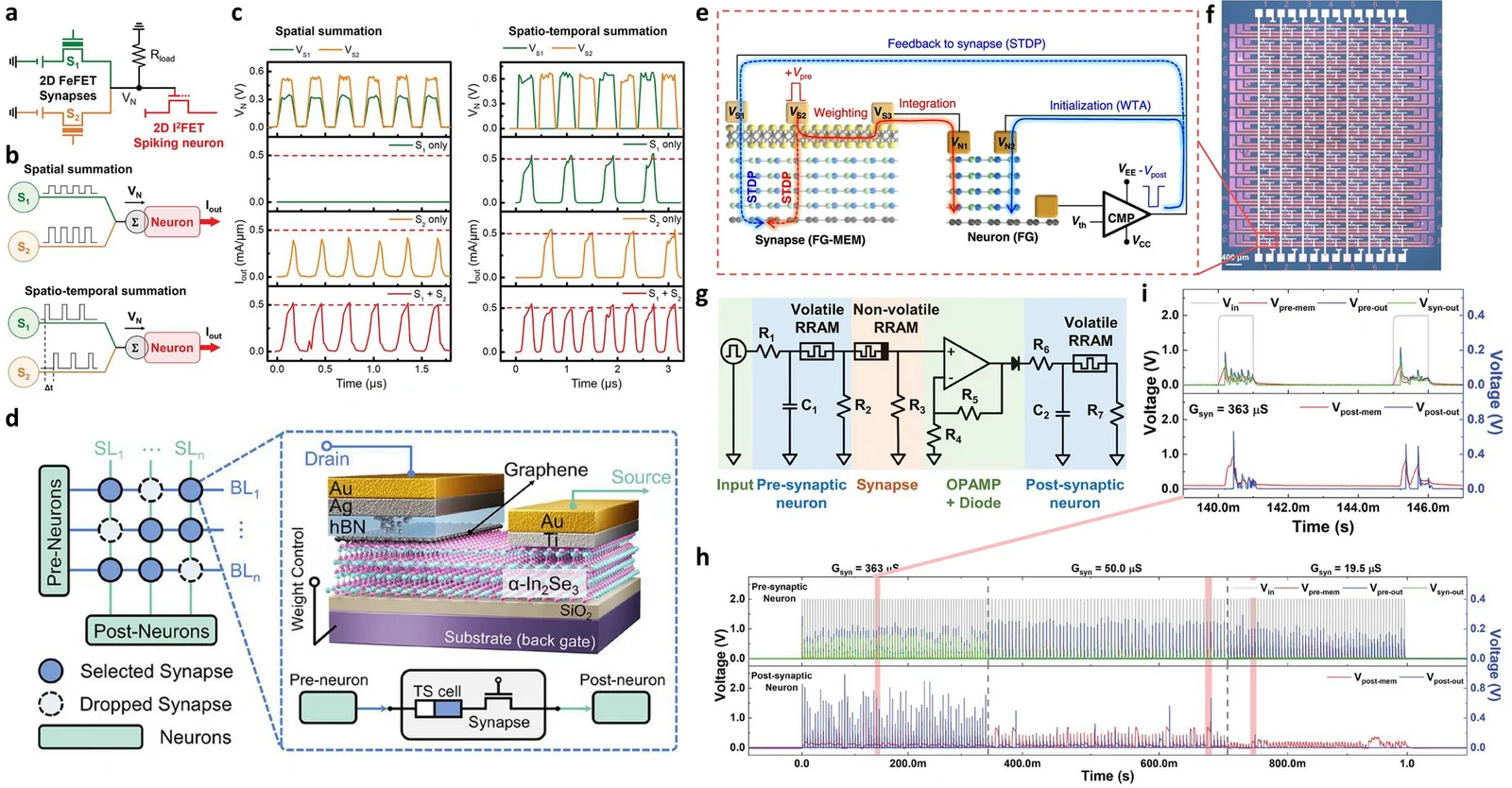
**a** Circuit schematic of an interconnected neural network comprising two synapses and one spiking neuron. **b-c** Schematic representation of the spatial summation and spatiotemporal summation of the two pulse inputs. The integrated spiking neuron responses are displayed in **c** Reproduced with permission [49]. Copyright (2024), Wiley–VCH GmbH. **d** Illustration of the DropConnect hardware implementation. Reproduced with permission [18]. Copyright (2025), Wiley–VCH GmbH. **e** Basic synapse–neuron assembly schematic demonstrating unsupervised learning through STDP synapses and LIF neuron functions. **f** Optical image of a large-scale integrated neurosynaptic array. Reproduced with permission [182]. Copyright (2023), Springer Nature. **g** Circuit schematic of a neuron–synapse–neuron interconnected hardware system. **h** Transient electrical monitoring of artificial neural networks under three distinct synaptic device conductance states. **i** Magnified view of the synaptic device conductance of 363 μS. Reproduced with permission [167]. Copyright (2023), Wiley–VCH GmbH

The interactions between artificial neurons and synapses constitute the core of neural information processing and network dynamic regulation, encompassing signal transmission, plasticity modulation, and network stability maintenance. Current research has identified three fundamental interconnection modes: (i) synapse toward neuron or neuron toward synapse information flow pathways. Synapses transmit information to modulate the activity of downstream neurons, or conversely, single-neuron impulses directly regulate synaptic weights, thereby establishing a directional information flow. For instance, distinct signals from 2D ferroelectric synapses are integrated by 2D impact ionization field-effect transistor (I^2^FET) neurons to emulate spatial and spatiotemporal summation (as illustrated in Fig. 11a–c) [49]. Zhou et al*.* also proposed a compact and energy-efficient interconnected architecture in which cycle-to-cycle variations in the TS voltage intrinsically induce stochastic synaptic weight decay (as demonstrated in Fig. 11d) [18]. In fact, the brain operates through coordinated activity in complex networks comprising hundreds of millions of neurons, which motivates the co-integration of synaptic devices and neuronal circuits on a single chip, enabling large-scale neuromorphic arrays capable of parallel computation. Notable progress was achieved by Yu’s group, who emulated neuronal membrane potentials using multi-terminal floating-gate memristors for interneuronal connections, achieving large-scale integration of a 7 × 16 crossbar array (Fig. 11f) [182]. Moreover, the network scale can be further expanded to execute complex cognitive tasks by cascading multiple chip modules through an expandable interconnected architecture. (ii) Neuron–synapse–neuron interconnection. Changes in synaptic plasticity through the activity of pre-neurons propagate to modulate distal neurons, establishing network-level adaptability. This effect was experimentally demonstrated by Jo et al*.* in Fig. 11g, h [167]. (iii) Interconnection feedback. Higher-order neuronal outputs modulate synaptic efficacy in primary neurons through feedback loops to establish dynamic equilibrium. As demonstrated by the neural unit circuit in Fig. 11e**,** the comparator switches from *V*_CC_ (0.01 V) to a *V*_EE_ (− 2 V) output upon receiving suprathreshold neural impulses. This signal not only serves to initialize membrane potentials in postsynaptic neurons but also provides feedback to presynaptic terminals for adaptive weight updates through STDP rules, thereby enabling online learning [182]. With continued advancements in materials, fabrication technologies, and device physics, the integration and interconnection of synaptic and neuronal devices is poised to achieve breakthroughs in functional complexity, energy efficiency, and biological fidelity.

### Multimodal Perception and Execution of Artificial Neurons and Synapses

**Fig. 12:**
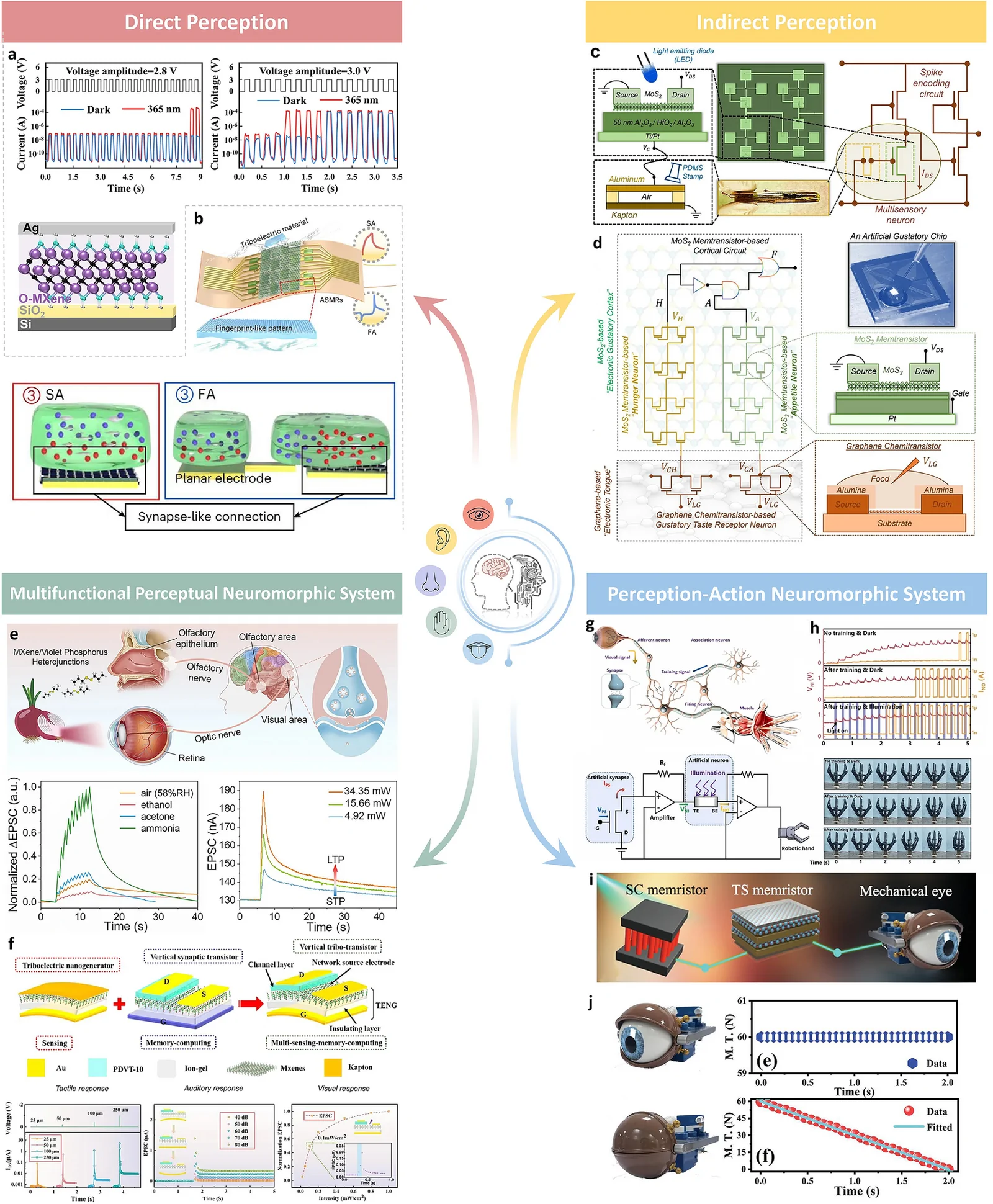
**a-b** Direct perception: **a** Device structure of oxidized MXene-based artificial optoelectronic memristor and integrate-fire behaviors respond to a series of electrical spikes with different voltage amplitudes. **b** Conceptual diagram of an artificial synapse based on an array with eight SA (slow-adapting) and eight FA (fast-adapting) mechanoreceptors and an enlarged structural schematic of the device with synaptic-like connections. Reproduced with permission [185]. Copyright (2025), Springer Nature. **c-d** Indirect perception: **c** Bio-inspired visuo-tactile multisensory neuron integrating a triboelectric tactile receptor and MoS_2_ optoelectronic memtransistor with spike encoding circuitry. Reproduced with permission [188]. Copyright (2023), Springer Nature. **d** A bio-inspired gustatory system based on graphene chemitransistor and MoS_2_ memtransistor for simulating psychological and physiological feeding behaviors. Reproduced with permission [189]. Copyright (2023), Springer Nature. **e**, **f** Multifunctional perceptual nervous system: **e** MXene/violet phosphorus heterojunction-based synapses for visual-olfactory cross-modal perception and PSC responses under varying gas environments and light intensities. Reproduced with permission [190]. Copyright (2024), Springer Nature. **f** Self-powered highly sensitive monolithic vertical transistor for tactile-auditory-visual multimodal perception and memory. Reproduced with permission [191]. Copyright (2022), Springer Nature. **g**, **h** Perception–action nervous systems: **g** Implementation of dynamic training processes in biological neural systems and biomimetic circuits. **h** Voltage and current responses in the circuit and corresponding robotic hand poses under three distinct training signals and optical signals. Reproduced with permission [61]. Copyright (2022), Elsevier. **i** An artificial vision system with interconnected photonic synapse, spiking neuron, and electronic eye actuator. **j** Tension in the electronic eye as a function of illumination duration under ordinary and bright light, with corresponding ocular states. Reproduced with permission [192]. Copyright (2022), Wiley–VCH GmbH

In the primary stage of biological systems receiving external data input, neurons integrate signals through two distinct modalities: responding to external stimuli directly through specialized neurons [183], and acquiring information indirectly via sensory cell interactions [184]. These dual pathways are precisely replicated and optimized in electronic systems. As specialized neurons shown by Zeng et al*.*, the excellent photoresponsivity of oxidized MXenes enables direct coupling with ultraviolet light to achieve optically assisted single-neuron spiking excitation, as illustrated in Fig. 12a [62]. Further advancing the direct perception, Fig. 12b showcases a direct perceptive synapse that natively integrates mechanoreceptive functionality [185]. The triboelectric gating effect, arising from direct or indirect contact between the receiving layer and the gate dielectric, modulates excitatory postsynaptic currents to enable both slowly adapting and rapidly adapting characteristics for signal preprocessing. Similar specialized neural devices also include thermally sensitive [186] and humidity-sensitive [187] devices, which directly integrate functional sensing 2D materials inside neuromorphic devices.

Some systems employ indirect perception. Sensors first transduce physical signals into electrical signals, which are then integrated by threshold neuron devices. Subsequently, it can be transmitted to the synapses for weight-update-dependent training signal propagation. Current research has achieved comprehensive coverage of five sensory modalities (auditory, olfactory, visual, gustatory, and tactile) by exploring 2D material heterointegration technologies, developing novel interconnection architectures, and incorporating the hierarchical organization and information processing principles of biological neural systems. These advances enable the emulation of increasingly sophisticated environmental adaptive behaviors. As displayed in Fig. 12c, d, Das’s team constructed circuits using graphene and MoS_2_ to emulate tactile and gustatory neural systems [188, 189]. The tactile study implemented mechanosensory neural coding through piezoresistive sensors coupled with synaptic transistors, while the gustatory investigation integrated chemitransistors with neuronal circuits to process both hunger (physiological) and appetite (psychological) signals.

Natural biological perception systems typically require simultaneous processing of multimodal stimuli and execute dynamic decision-making through sophisticated cooperative regulation. In Fig. 12e, Ma et al*.* report an optoelectronic synapse based on the persistent photoconductivity effect in MXene/violet phosphorus heterojunctions, capable of emulating diverse synaptic behaviors [190]. The device exhibits distinct optoelectronic responses as well as image learning and memory characteristics in certain chemical environments, achieving synaptic-level simulation of visual-olfactory cross-modal perception. In the study by Liu et al*.*, triboelectric potentials, acoustic waves, and optical stimuli were transduced into unified PSC through modulation of the electrical double layer in ion gels and the Schottky barrier at MXene/semiconductor interfaces, ultimately enabling multimodal emotion recognition as shown in Fig. 12f [191].

Furthermore, the interconnected control between neuromorphic systems and actuators enables closed-loop intelligent behaviors spanning perception to action. Such systems typically process information through neuromorphic devices, then drive actuators by spike-encoded outputs, while employing learning rules (e.g., STDP and BCM) to allow dynamic movement strategy adjustments akin to biological nervous systems. Typical applications include an integrated visual perception-actuation (Fig. 12g) system to emulate the hand-withdrawal reflex. The output of the system drives a bio-inspired actuator under three operational conditions, with the activation time synchronized to spike emission. With training and illumination, the activation time decreases significantly, and the retraction response becomes more pronounced (Fig. 12h) [61]. The electronic eye system developed by Yan’s team primarily utilizes Sb_2_Se_3_/CdS-core/shell nanorod array optoelectronic memristor as photonic synapses [192] (Fig. 12i). Synaptic weight updates modified by light intensities induced corresponding adjustments in both the spiking frequency and amplitude of neuronal outputs. Under high-intensity illumination, actuator tensile force decreases to trigger eyelid closure for light attenuation, whereas normal light conditions maintain eyelid openness for optical information acquisition (Fig. 12j). These advancements are propelling perception-actuator systems beyond simple motion mimicry toward autonomous intelligent behaviors, thereby establishing novel design paradigms for next-generation robotics, intelligent prosthetics, and adaptive mechanical systems.

### Neuromorphic Hardware Systems for SNNs

**Fig. 13:**
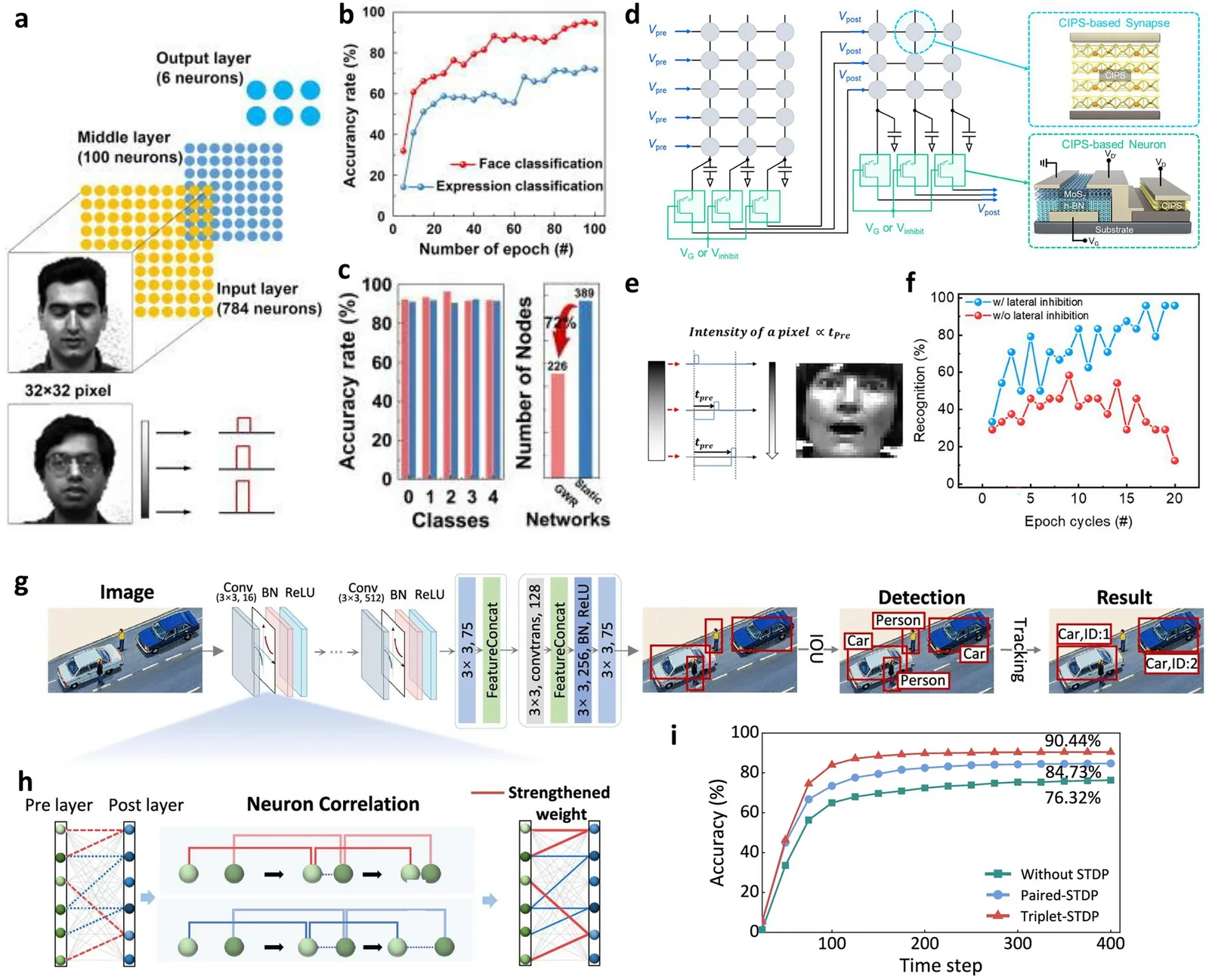
**a** Schematic of a three-layer spiking neural network for facial and expression classification. **b** Facial and expression recognition accuracy after 100 training epochs. **c** GWR network dynamically reduces node count by 72% with superior efficiency. Reproduced with permission [10]. Copyright (2023), AIP Publishing. **d** A neuron- and synapse-based pseudo-crossbar array for SNN implementation. **e** Intensity of input pixels encodes the timing of presynaptic spikes. **f** Recognition rate with and without lateral inhibition across training epochs. Reproduced with permission [69]. Copyright (2025), Wiley–VCH GmbH. **g** YOLO-SNN architecture schematic and workflow for dynamic object detection and tracking. **h** Event correlation-dependent plasticity process in the triplet-STDP learning rule. **i** Tracking accuracy comparison of the triplet-STDP-enabled SNN, paired-STDP-enabled SNN, and the original YOLO-SNN. Reproduced with permission [199]. Copyright 2025, Springer Nature

The neuroscience-oriented spiking neural network (SNN) is ideal for event-driven applications and can be deployed on neuromorphic chips for parallel, low-power operation. Advancements in neuromorphic hardware are significantly expediting the deployment of SNNs, thereby expanding the frontiers of AIoT. Neuromorphic hardware systems employing 2D materials as functional layers demonstrate distinct advantages in pattern recognition applications: (i) Relatively high charge carrier mobility enables rapid data processing and transmission during pattern recognition tasks to enhance computational efficiency [193–195]. This is necessary for applications that require real-time decision-making [196]. (ii) Exceptional mechanical robustness enables systems to maintain reliable performance under repeated dynamic deformation, suitable for SNN systems in biomimetic robotic surfaces and wearable health monitoring applications [197, 198]. (iii) The tunable material properties provide a physical foundation for the precise mapping of SNN learning rules (e.g., STDP) at the hardware level [122, 199]. In recent years, image computing platforms leveraging 2D materials have been continuously explored and innovated. In Fig. 13a a three-layer SNN was designed for processing the Yale Face Database, where pixel values were converted into stochastic spike voltages through temporal coding, with synaptic and neuronal circuits performing subsequent information processing. The results indicate that the SNN achieves accuracies of 71.6% and 95.8% for facial expression recognition and face classification tasks, respectively (Fig. 13b, c). The system is further applicable to recognition tasks in adaptive dynamic neural networks (Growing When Required, GWR) [10]. Figure 13d exhibits a crossbar array based on CuInP_2_S_6_ -based synaptic and neural devices. This SNN achieves unsupervised learning by engineering targeted temporal correlations between presynaptic and postsynaptic spikes. The detected pixels are converted into presynaptic spikes using pulse delay timing to encode the analog information of pixel intensity (Fig. 13e). Following 20 training epochs with the implementation of the lateral inhibition function in neurons, a high recognition accuracy of 95.83% was achieved (Fig. 13f) [69]. Recent advances demonstrate that SNN architectures employing diverse learning rules are evolving from static image analysis to complex dynamic vision processing, marked by the addressing of critical challenges such as real-time object detection and tracking for autonomous driving. A vehicle tracking application based on the triplet-STDP-enabled YOLO-SNN was realized by Zhang’s team. Efficient feature extraction relies on the weight update process through pairwise correlations between neurons in the preceding layer (Fig. 13g–h). Even under overlapping conditions between two target vehicles, the network equipped with triplet-STDP maintains precise tracking, achieving a detection accuracy of 90.44% (Fig. 13i) [199].

### Neuromorphic Devices for Logical Operations

**Fig. 14:**
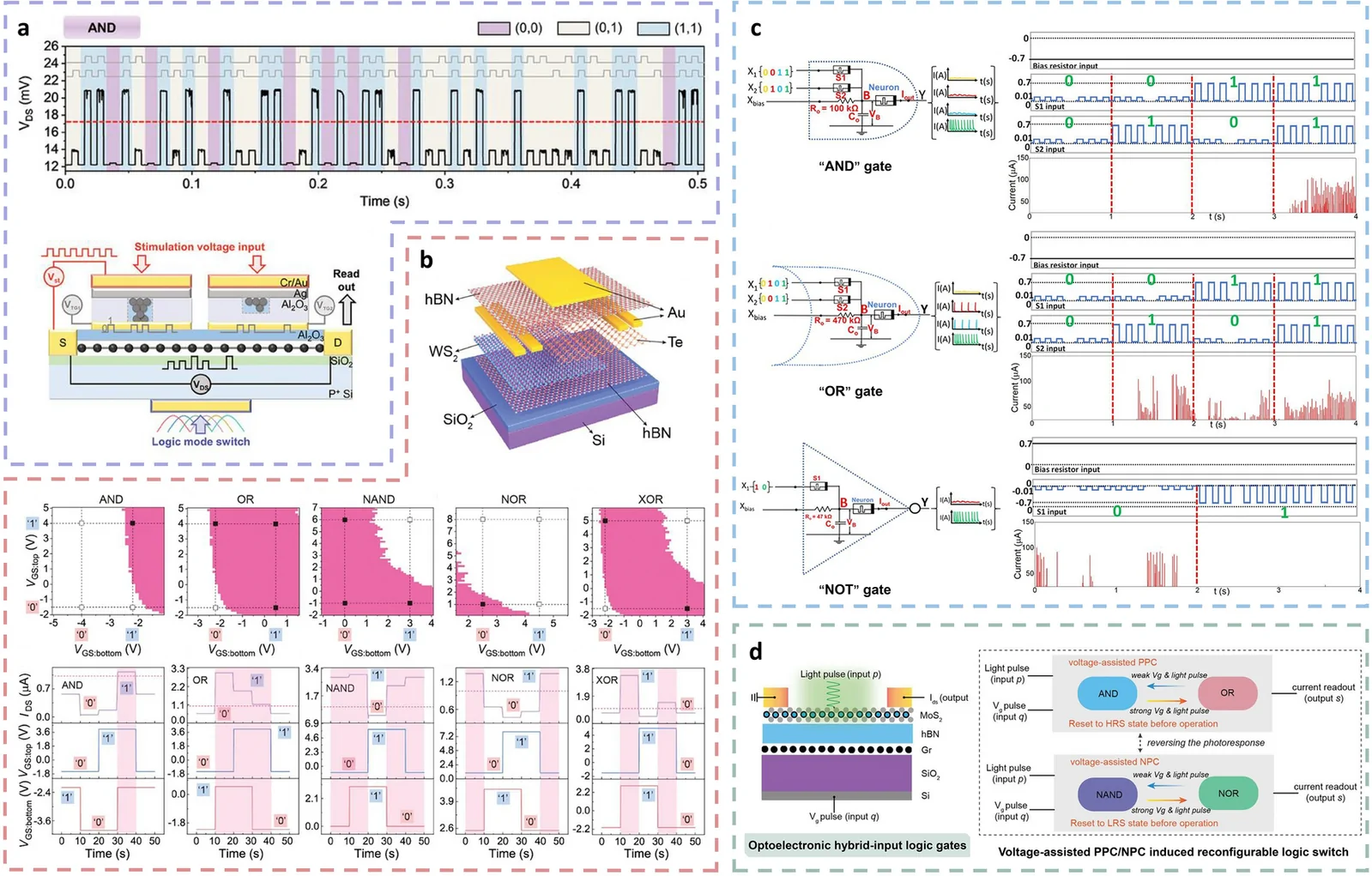
**a** Schematic of the neuron transistor’s logic operations, working principle, and an “AND” logic mode implemented through spatiotemporal integration. Bottom-gate architecture for multi-logic mode switching. Reproduced with permission [19]. Copyright (2024), Wiley–VCH GmbH. **b** An exploded view of the device configuration based on Te/WS_2_ heterojunction and five distinct logic modes implemented in a single device through synergistic top-gate and bottom-gate control. Reproduced with permission [200]. Copyright (2024), Wiley–VCH GmbH. **c** Implementation schematics of two-input AND, OR, NOT logic gates and corresponding spiking outputs post-neuronal integration. Reproduced with permission [201]. Copyright (2022), American Chemical Society. **d** Diagram of optoelectronic hybrid-input logic gates and operational scheme illustration of reconfigurable non-volatile optoelectronic logic modes. Reproduced with permission [202]. Copyright (2022), Wiley–VCH GmbH

Traditional CMOS logic gates are widely employed in microprocessors and microcontrollers. The basic logic gate, such as a NOT gate, requires at least one NMOS and one PMOS transistor connected in series. Implementing more complex combinational and sequential logic gates necessitates additional transistors, bypass capacitors, and multi-level cascading circuits, resulting in significant circuit complexity. Consequently, there are pressing needs for novel approaches that balance high integration density, computational efficiency, and precise temporal control. Notably, artificial neurons and synapses can be directly utilized as fundamental physical units for the execution of logical operations. When the weighted sum of two input signals exceeds the rated threshold, the neuron fires suprathreshold spikes (logic “[1, 1]”); otherwise, it remains in a resting state (logic “[0,1]” or “[0,0]”), as illustrated in Fig. 14a [19]. Memristive synapses, leveraging their dynamic resistive characteristics, can directly realize hardware-level logic state storage and sequential calculation through gating control. This intrinsic programmability enables their configuration as NAND, OR, XOR, and other logic gates, as demonstrated in Fig. 14b [200]. Roy’s team proceeds to connect multiple synapses and biased resistors to govern neuronal output currents to implement AND, OR, and NOT Boolean logic gate functionalities in a monolithically integrated circuit, as shown in Fig. 14c [201]. Furthermore, numerous neuromorphic hardware platforms exhibit multimodal tunable logic capabilities. A representative example is optoelectronic co-control, illustrated in Fig. 14d, where Yang et al*.* proposed a reconfigurable Boolean logic scheme [202]. Relying on the device’s positive (PPC) and negative (NPC) photoconductance effects, the system demonstrates four distinct logic states: AND and OR in PPC mode, alongside NAND and NOR in NPC mode through utilizing voltage polarity- and light intensity-encoded binary conductance switching. Programmable logic functionalities can be further expanded through electrical, optical, and mechanical multi-stimuli modulation strategies [203–207].

## Roadmap and Challenges

**Fig. 15:**
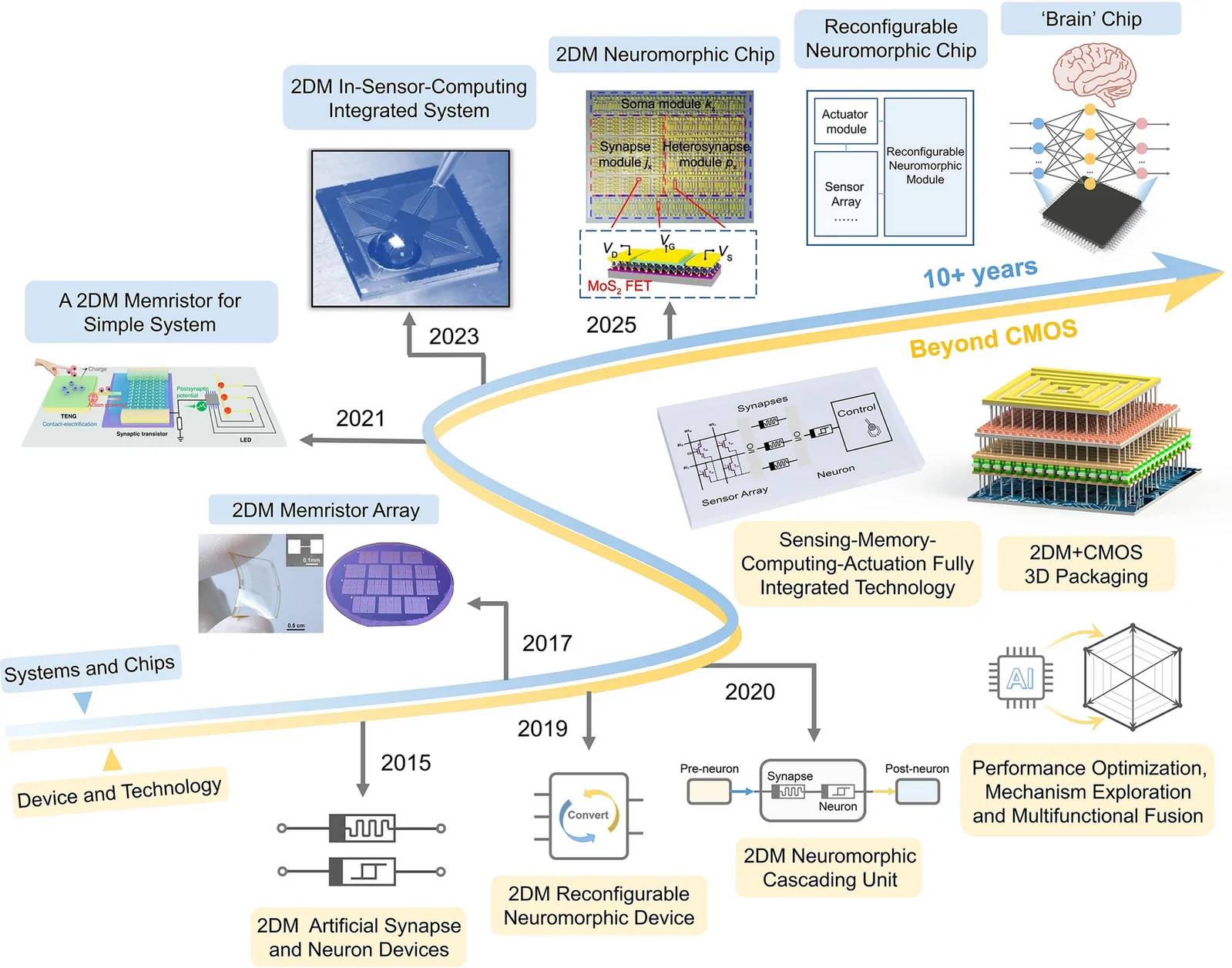
Roadmap of the 2D material neuromorphic device toward a chip. Reproduced with permission [211]. Copyright (2017), Wiley–VCH GmbH. Reproduced with permission [212]. Copyright (2021), Springer Nature. Reproduced with permission [213]. Copyright (2021), Springer Nature. Reproduced with permission [189]. Copyright (2023), Springer Nature. Reproduced with permission [210]. Copyright (2025), Springer Nature. Reproduced with permission [5]. Copyright (2024), Springer Nature

2D material-based neuromorphic device engineering and chip prototyping is a nascent and rapidly evolving frontier. As shown in Fig. 15, the development track began with 2D memristors and has since evolved into diverse platforms based on various mechanisms, successfully emulating biological synaptic and neuronal functionalities. Reconfigurable neuromorphic devices subsequently emerge as an ingenious and pivotal solution to the challenges of the post-Moore era. With continuous process advancement and technological refinement, research has evolved from individual device units to array integration and system-level demonstrations. In recent years, cutting-edge research has increasingly focused on sensor-in-computing and the monolithic integration of neuromorphic modules. Notable milestone breakthroughs in this field encompass: a MoS_2_/Ag nanograting optoelectronic transistor array capable of simultaneous sensing, preprocessing, and recognizing optical images without latency [208]; a 16 × 16 computing kernel based on 2T-2R unit with 3D heterogeneous integration [209]; and integration of MoS_2_-based reconfigurable transistors into neuromorphic systems as synaptic, heterosynaptic, and neuronal soma modules [210]. Future research on neuromorphic devices and systems should focus on key directions such as performance optimization, mechanism exploration, multifunctional fusion, and advanced integration technologies, among others. AI technologies can be leveraged to assist in material library expansion, process parameter optimization, and functional device design, thereby enhancing the biological plausibility and overall performance of devices. Through three-dimensional, high-density integration of 2D materials with CMOS, future neuromorphic systems are expected to achieve more efficiencies in terms of area and a substantially larger integration scale. Building upon this foundation, neuromorphic chip architectures with dynamic reconfiguration capabilities can be progressively developed, ultimately leading to brain-like chips that rival biological systems in perception, information processing, and adaptive computation.

**Fig. 16:**
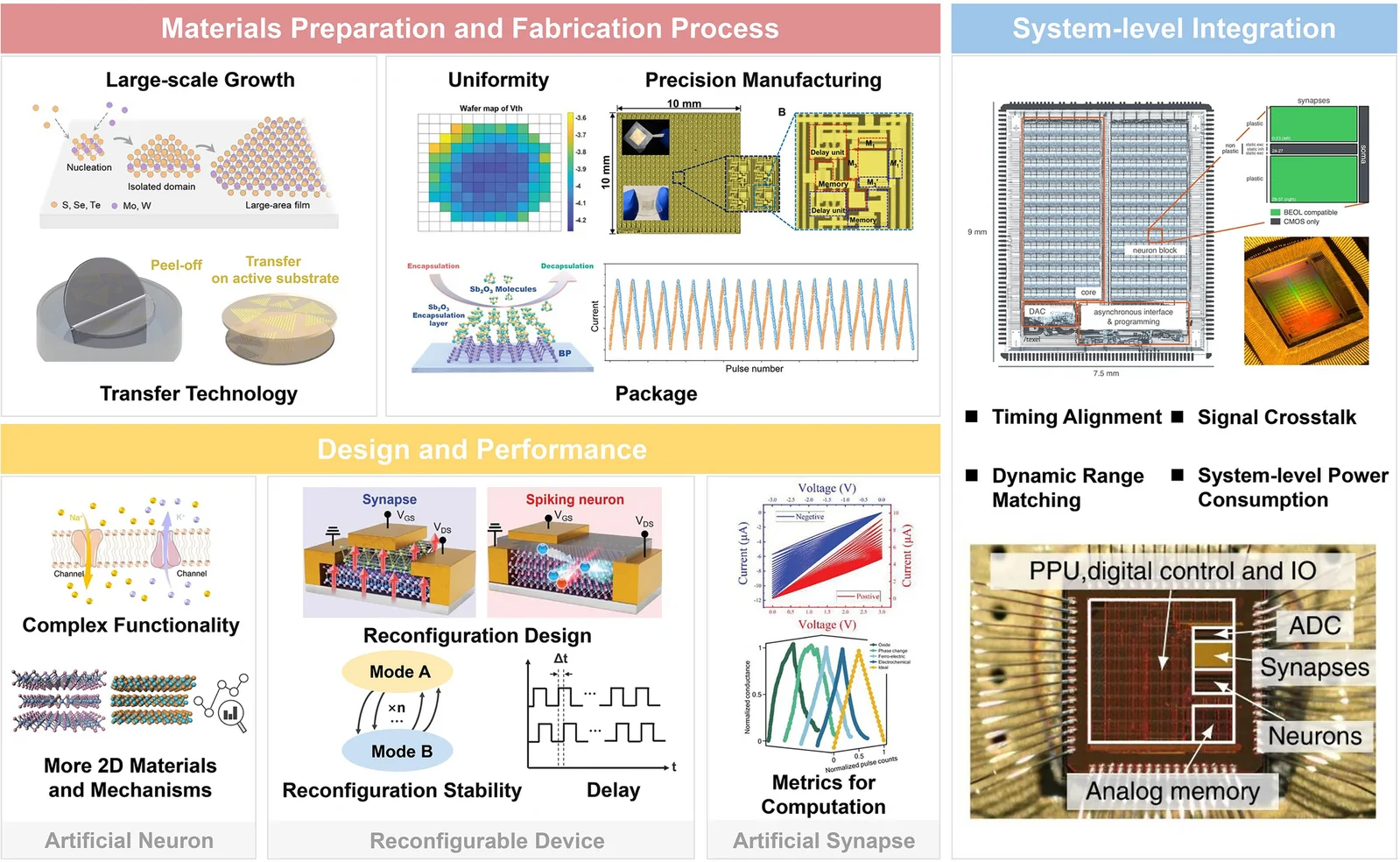
Challenges of 2D materials in neuromorphic devices and systems. Reproduced with permission [227]. Copyright (2024), American Chemical Society. Reproduced with permission [223]. Copyright (2025), American Chemical Society. Reproduced with permission [228]. Copyright (2021), IEEE. Reproduced with permission [229]. Copyright 2022, American Association for the Advancement of Science. Reproduced with permission [225]. Copyright (2022), Wiley–VCH GmbH. Reproduced with permission [16]. Copyright (2025), Springer Nature. Reproduced with permission [76]. Copyright (2022), Springer Nature. Reproduced with permission [230]. Copyright (2017), Wiley–VCH GmbH. Reproduced with permission [49]. Copyright (2024), Wiley–VCH GmbH. Reproduced with permission [231]. Copyright (2025), AIP Publishing. Reproduced with permission [232]. Copyright (2024), Wiley–VCH GmbH. Reproduced with permission [233]. Copyright (2025), Springer Nature. Reproduced with permission [234]. Copyright (2020), Springer Nature

However, there is still a significant gap between the demonstrated neuromorphic hardware system and those needed for practical large-scale computing applications. A series of barriers remains to be overcome to approach the complexity and reliability of biological systems. The challenges for further research in this field mainly lie in three aspects: (i) material preparation of 2D materials and fabrication process; (ii) design and performance at the device level, and (iii) integration for system-level applications. A schematic illustration is summarized in Fig. 16.

### Material Preparation and Fabrication Process

The fabrication of high-quality 2D semiconductor materials serves as the fundamental basis for advancing them toward integrated circuit applications. In current single-device demonstrations, top-down approaches, such as mechanical exfoliation, obtain high-quality few-layer materials. However, these approaches exhibit extremely low yield, uncontrollable dimensions, incapability of precise patterning, and significant performance fluctuations [214, 215]. Prior research has demonstrated that chemical vapor deposition (CVD, a bottom-up approach) can achieve wafer-scale growth [216–218], but this method is plagued by certain limitations such as grain boundary defects in polycrystalline films, limited single-crystalline domain sizes, doping impurities, and difficulty in controlling the nucleation [219]. Recent studies have reported promising technological advances. The rapid growth of centimeter-scale monolayer MoS_2_ single-crystal domains has been achieved using a confined growth method with two-dimensional molten precursors [220]. Separately, a solid–liquid–solid strategy enables conversion of amorphous InSe films into pure-phase and highly crystalline InSe sheets with uniform coverage across ~ 5 cm wafers [221]. To meet the demands of diverse substrates, some transfer strategies that utilize the surface tension of solutions and liquid nitrogen-assisted stripping are gradually replacing etching techniques in pursuit of cleaner and damage-free transfers [222–224]. Certainly, these growth and transfer technologies are yet to mature for the transition from laboratory to fabrication. Beyond materials growth and transfer, systematic considerations of uniformity and stability in the device fabrication process remain imperative. For instance, in simulating synaptic weight updates, the global and local variations caused by non-uniform layer growth (e.g., thickness, defects, and interaction area) or etching across wafers lead to fluctuations in resistive switching behavior, degrading device-to-device uniformity. Moreover, with progressive device miniaturization, linewidth variation and overlay error emerge as critical concerns, which directly induce fluctuations in the effective channel size and increased parasitic contact resistance. During the integration process, conventional semiconductor processes involve critical steps such as thermal oxidation, annealing, and plasma etching, which can easily induce material decomposition, interface contamination, and even structural damage in 2D materials. These impairments subsequently lead to signal attenuation and timing jitter, thereby further disrupting the emulation of neural behavior. Equally noteworthy is the intrinsic air instability of two-dimensional materials. Exposure to humid and oxygen-containing environments can directly induce material oxidation, increase defects and surface adsorption, leading to uncontrolled drift and degradation of intrinsic electrical properties such as carrier mobility, bandgap, and doping type. The heat accumulation in neuromorphic devices under high-frequency pulsed stimulation will accelerate this process. These constraints result in threshold voltage fluctuations and a reduction in switching ratios under cyclic operation (cycle to cycle), which prevents the reliability criteria demanded in implementations. Therefore, efficient encapsulation strategies constitute critical prerequisites for achieving stable operation in 2D material-based neuromorphic devices. Van der Waals (e.g., h-BN), atomic-layer-deposited (e.g., Al_2_O_3_), and polymer-based (e.g., PMMA) encapsulation approaches can significantly suppress environmental degradation of 2D materials by employing physical barrier layers and interface passivation. Nevertheless, it is worth considering that such encapsulation strategies must ensure long-term environmental stability while avoiding the introduction of additional defects or mechanical stress, so as to preserve the intrinsic performance of the material [225].

### Design and Performance of Neuromorphic Devices

Research on synaptic devices has now established a relatively diverse materials and regulatory strategies. Nevertheless, research efforts should continue to focus on critical characteristics such as linear and symmetric weight update, dynamic range, and resolution, as these performance metrics crucially determine the computational accuracy and efficiency of neural networks. Moreover, compared with the flourishing progress in 2D material-based synaptic devices, the development of neuronal devices still lags in terms of performance and 2D material exploration. Neuronal devices impose more complex functionality requirements, which necessitate simultaneous fulfillment of threshold characteristics, signal integration/firing, refractory period properties, and cross-modal perception, among others, to emulate biological neuron functionality, an area where research remains insufficient. Neuronal intrinsic plasticity plays a fundamental role in regulating complex brain functions, primarily through three pivotal mechanisms: the adjustment of spike threshold, the amplification of excitatory postsynaptic potentials, and changes in resting potential. Nevertheless, research exploration in neuronal intrinsic plasticity remains at a preliminary stage [53, 226]. On the other hand, the potential of emerging 2D materials for artificial neurons remains underexplored, and the correlation mechanisms between material properties and desired neuronal functionalities lack systematic investigation. Further screening of 2D materials (e.g., ferroelectric and phase-change material systems) with intrinsic threshold characteristics and fast dynamics can be achieved by integrating theoretical calculations (density functional theory, molecular dynamics simulations) with experimental validation. Alternatively, controlled defects, heteroatom introduction, and interface engineering in 2D materials can be utilized to adjust carrier mobility and energy barriers, thereby emulating neuronal refractory periods and firing threshold behaviors.

In terms of reconfigurable devices, a central challenge lies in the design of devices. Exploring how to systematically integrate emerging heterogeneous materials, multiple physical mechanisms, and structure design to synergistically optimize device performance and reconfigurability is the core research direction in the next stage. In addition, the physical switching mechanisms of functional materials (e.g., domain wall polarization reversal or conductive filament formation/rupture) are often constrained by their intrinsic kinetic processes. Consequently, undesirable operational delays ranging from microseconds to milliseconds arise during reconfiguration, making the device mode transitions incompatible with the real-time requirements of neuromorphic computing. This might require modulating the dynamic process or relying on error-correction coding techniques and algorithms for compensation. Despite current studies having extensively validated the endurance of neuromorphic devices in single-modal operations such as synaptic plasticity or spike triggering, their reconfiguration stability under high-frequency switching conditions, including materials degradation, cycling stability, and state retention capabilities, still lacks systematic verification. Subsequent efforts must focus on developing evaluation standards for reconfiguration stability while ensuring robustness through material stability, encapsulation, and heat dissipation. Moreover, reconfigurable devices may exhibit performance fluctuations due to challenges in coordinating their internal mechanisms. The optimization can be carried out in three aspects. The first approach involves setting precise switching thresholds for different modes and employing multi-port/multi-dimensional heterostructures to physically separate the mechanisms. Operationally, it is essential to ensure that the device is allowed sufficient relaxation time before functional reconfiguration to eliminate the residual effects of its prior state. Third, feedback and calibration circuitry can be introduced to dynamically monitor and compensate for parameter drift. Notably, although reconfigurable devices exhibit significant potential in reducing size and enhancing adaptiveness, this approach entails sacrificing certain extreme performance and energy efficiency, which represents a trade-off strategy. Consequently, in scenarios where functional requirements are clearly defined, demands remain stable, and extreme performance or energy efficiency is essential, dedicated devices continue to hold distinct advantages. Future systems are likely to combine both approaches to fulfill diverse application requirements: a reconfigurable hardware layer will be responsible for task scheduling, dynamic adaptation, and preliminary processing, whereas a dedicated hardware layer will handle computationally intensive and highly optimized core tasks.

### System-level Integration

The integrated neuromorphic hardware systems require careful consideration across multiple domains, including dynamic range matching, timing alignment, system-level power consumption, and signal crosstalk. 2D material-based devices may exhibit characteristic mismatches (e.g., voltage/current levels) with silicon CMOS circuits, necessitating additional interface circuitry that increases system complexity and power consumption. Timing misalignment manifests primarily as signal delay mismatch, caused by suboptimal carrier mobility in certain 2D materials [194], interconnect resistance–capacitance (RC) delays, and stochastic device switching speeds. Such timing deviations disrupt precise time-dependent relationships in SNNs, particularly STDP learning, and can induce erroneous synaptic weight updates and computational errors. Neuromorphic computing systems must rigorously address power consumption as a fundamental design constraint, which originates from static power dissipation due to leakage currents in high-density integration, dynamic response power during event-driven operation, system-level power demands from synaptic-neuron communication, as well as additional power overhead for active thermal management. Regulation systems operating within a multi-physics field need to consider signal crosstalk and decoupling strategies carefully.

To sum up, many challenges exist in building more powerful artificial neurons, synapses, and integrated systems based on 2D materials. Future breakthroughs and even industrialization implementation are contingent on a market-guided, synergistic innovation across the entire chain. This necessitates coordinated progress spanning the controllable preparation and process optimization of high-quality 2D materials, innovations in device design, and advances in high-density heterogeneous integration, to the construction of a co-designed hardware-software computing architecture. A closed-loop iteration and continuous optimization encompassing materials, devices, integration, and system algorithms will ultimately pave the way for a transformation in brain-inspired chips and memory architectures.
